# Subcutaneous hydration and medications infusions (effectiveness, safety, acceptability): A systematic review of systematic reviews

**DOI:** 10.1371/journal.pone.0237572

**Published:** 2020-08-24

**Authors:** Daphne Broadhurst, Marie Cooke, Deepa Sriram, Brenda Gray

**Affiliations:** 1 Alliance for Vascular Access Teaching and Research (AVATAR) group, Menzies Health Institute Queensland, Queensland, Australia; 2 Infusion Excellence Consulting, Ottawa, Canada; 3 School of Nursing and Midwifery, Nathan Campus, Griffith University, Queensland, Australia; 4 Clinical Pharmacy Partners, Tampa, Florida, United States of America; University of Porto, PORTUGAL

## Abstract

**Objective:**

To synthesize the current evidence for subcutaneous hydration and medication infusions from systematic reviews and to assess their methodological quality.

**Introduction:**

Peripheral intravascular cannula/catheter insertion is a common invasive procedure for administering fluids and medications. Venous depletion is a growing concern for several patient populations. Subcutaneous access for the administration of isotonic solutions and medications is an alternative; however, vascular access assessment and planning guidelines rarely consider this route.

**Methods:**

Systematic review of systematic reviews (PROSPERO CRD42018046504). We searched 6 databases published in English language from 1990 to June 2020, identifying subcutaneous infusions an alternate route for fluids or medication. Methodological quality was evaluated using AMSTAR 2 criteria and data for mechanisms of infusion and outcomes related to effectiveness, safety, efficiency and acceptability extracted. The Johanna Briggs Institute’s grades of recommendation informed the strength of recommendation.

**Results:**

The search yielded 1042 potential systematic reviews; 922 were excluded through abstract and duplicate screen. Of the remaining articles, 94 were excluded, and 26 were included. Overall, evidence is strong for recommending subcutaneous hydration infusions for older adults, weak for pediatric patients and inconclusive for palliative patients. There is strong evidence for 10 medications; weak evidence supporting 28 medications; however, there are eight medications with inconclusive evidence to make a recommendation and four medications not appropriate for subcutaneous delivery.

**Conclusion:**

Subcutaneous access should be considered alongside intravenous therapy for hydration in older adults, and several medications. There are additional benefits in terms of ease of use and cost-effectiveness of this mode. Inclusion of subcutaneous access in clinical guidelines may promote uptake of this route to help preserve vessel health of vulnerable patients. Further high-quality research is needed to inform subcutaneous infusion therapy in a variety of populations (including pediatrics and palliative care) and medications and clarifying the mechanism of delivery.

## Introduction

Infusion therapy is a common treatment modality to deliver medications and fluids in the acute and home care settings and is gaining prevalence in the long-term care setting. Traditionally, these parenteral therapies have been delivered via the intravenous route. However, venous depletion is a growing concern with an increasing aging population and patients with long-term complex co-morbidities [1–3]. The problems of venous depletion are compounded by unnecessary peripheral venipuncture which add to patient physical and psychological trauma, compromised intravenous-related outcomes, suboptimal use of healthcare resources and increased costs [2, 4–7]. Additionally, infusion therapy is moving beyond the boundaries of the acute care and home care sector, to hospices and long-term care facilities [8]. Recently published vascular access planning tools are either oriented primarily to the acute care setting or do not address the option of subcutaneous access [9, 10].

The Infusion Therapy Standards of Practice recommend consideration of subcutaneous access for the administration of isotonic solutions and for continuous opioid and other infusion therapies/medications (e.g., immunoglobulin therapy) [11]. Subcutaneous access is achieved through the placement of a small catheter in the subcutaneous tissue, with the infusate absorbed from this space into the circulatory system. This route has the advantages of requiring a small catheter and less technical insertion skills, than that used for intravenous access, with its ease of application lending to its use in multiple settings [12]. Additionally, time to place the catheter is less and fewer complications are likely resulting in cost benefits [13, 14]. Although practiced since 1865, there has been a slow uptake, in part due to a lack of familiarity with the technique among physicians and healthcare professionals and perceived suboptimal outcomes (e.g., hypovolemic shock) due to inappropriate use of hydration solutions [12, 13]. In our scoping literature search to prepare for a systematic review of primary studies, numerous systematic reviews were identified. However, each either had a narrow scope addressing one treatment (e.g., Fisher and colleagues’ review of iron overload management, [15]) or provided limited literature search or limited evidence (e.g., Duems-Noreiga and Blasco’s [16] review of subcutaneous fluid and drug delivery).

Given the world’s aging population, health care systems rapidly changing, diversity in practice setting, and complexity of care and patient health conditions, the uptake and use of subcutaneous continuous infusions may help address these issues and challenges. The aim of our study is to determine the effectiveness, safety, acceptability and efficiency related to the use of subcutaneous infusion (SCI) as an alternate route to intravenous for the management of conditions or treatments such as dehydration and palliation, for children and adults in all care settings through a synthesis of systematic reviews of studies. A secondary objective is to identify the mechanisms of subcutaneous fluid and drug delivery that facilitated achieving these outcomes. This will provide an up-to-date and rigorous review of subcutaneous infusion therapy.

## Material and methods

We adopted the systematic review of systematic reviews [17] methodology from the Joanna Briggs Institute’s to conduct this review [18]. The study protocol was registered with the PROSPERO database (registration number CRD42018046504) and we report the review according to the Preferred Reporting Items for Systematic Review and Meta-analysis (PRISMA) guidelines (S1 Table) [19].

### Eligibility criteria

We considered all types of systematic reviews which included the following characteristics:

- A clearly stated set of objectives with an explicit, reproducible methodology;
- A systematic search that attempts to identify all studies that would meet the eligibility criteria;
- An assessment of the validity of the findings of the included studies (e.g., assessment of risk of bias and confidence in cumulative estimates); and
- Systematic presentation, and synthesis, of the characteristics and findings of the included studies, +/- meta-analyses [19].

Where some of the primary studies in the reviews were duplicated, we did not exclude the reviews, as they had different aims and objectives and thus added to the overall understanding of SCI to meet the aims of this current systematic review. We did however only include the primary studies’ findings once in our analysis and synthesis. Expert opinion/consensus and bench research, abstracts, and editorials/correspondence were excluded [18]. No restrictions regarding age, gender, diagnosis, geographical location, or healthcare setting were applied.

We included reviews that assessed interventions that used subcutaneous infusion (for a duration of around 2 hours or more) as an alternate route for fluid or medication therapy. Subcutaneous infusion is defined as the delivery of fluids or medication into the subcutaneous space for absorption into the circulation via “perfusion, diffusion, balance between hydrostatic/osmotic pressure and lymphatic drainage” ([16], p.118). An infusion of around 2 hours duration was determined from considering the 21 studies in a review by Caccialanza et al. [12], where the range of infusion was 2 hours to greater than 5 days. During the full text screening, numerous intermittent SC insulin vs continuous SC insulin systematic reviews were identified and excluded because they did not meet our inclusion criteria of SCI as an alternate route. Reviews that included other routes as comparators (such as intravenous and intraosseous) were excluded if data on subcutaneous infusions could not be extracted separately.

### Outcome measures

The primary outcomes of interest investigated included:

- Effectiveness: defined as clinical response to therapy (e.g., cure/improved, clinical failure or no change; completion of therapy within prescribed time frame);
- Safety: defined as medication or vascular access adverse event (e.g., abscess, erythema, bruising, electrolyte imbalance, edema, infection, pain, fluid overload, vascular collapse, and route failure), survival status (e.g., died of underlying condition, other causes, lost to follow-up or status unknown) and complications related to treatment (e.g., unplanned hospital readmission related to treatment;
- Acceptability: defined as patient and/or health care provider preference, satisfaction or perceived benefits of subcutaneous therapy;
- Efficiency: defined as healthcare resource utilization, including costs of infusion therapy supplies and treatment time.

Secondary outcomes included: indications for subcutaneous infusion therapy; medication/ solution type; infusion rates, volumes and duration; subcutaneous access sites; dwell times; and infusion control devices used.

### Search strategy

A systematic search was conducted November 2018 and updated in June 2020 of reviews from 1990 (as recommended by Aromataris et al. [18]) from the following databases: Excerpta Medica database (Embase), PubMed, Cumulative Index to Nursing and Allied Health Literature (CINAHL), Cochrane Database of Systematic Reviews, Joanna Briggs Institute of Systematic Reviews, and Database of Abstracts of Reviews of Effects (DARE). Search terms included combinations of Medical Subject Headings (MeSH) and key word terms [‘Subcutaneous Infusion’ OR Hypodermoclysis OR ‘Subcutaneous therapy’] and the term ‘Systematic Review’, with the assistance of a university librarian. Study resources limited inclusion to only English language (as listed in S2 Table). One author of a relevant abstract was contacted to establish the status of the review [20]; however, the review had not been completed.

The title and abstract of each article were scanned (independently by one reviewer: DS) and full copies of articles of potentially eligible reviews were obtained. Full texts of these reviews were then screened independently by two reviewers (MC and DB) against the review selection criteria. Disagreements were resolved by discussion between these reviewers and with consultation by a fourth reviewer and subject expert (BG).

### Data extraction and quality appraisal

Data were extracted and assessed independently by the reviewers in pairs ([BG, DB]; [MC, DS]), with anomalies reconciled by agreement. Data were obtained primarily from the systematic reviews, although primary studies were consulted for critical missing data (e.g., missing infusion properties). Data extracted included author/year, aim, design and number of studies included, search strategy, population, intervention/comparator, quality appraisal of primary studies, infusion characteristics, outcomes as previously defined, key findings, study limitations, funding, and conclusions.

Methodological rigour of each review was independently assessed by four reviewers in pairs as above, using the AMSTAR 2 tool [21]. This is a 16-item tool used to appraise the quality of systematic reviews and is frequently used in Cochrane overviews [17]. A score of overall confidence (high, moderate, low and critically low) was assigned by the reviewers to depict the accuracy and comprehensiveness of the data summary and critical methodological flaws [21]. This assessment informed the final grading of the recommendations.

### Data analysis and synthesis

Due to the different interventions, outcomes and outcome measures, a meta-analysis was not feasible. Quantitative outcome data are provided and synthesized where possible. Only data from randomised controlled trials (RCTs) and prospective cohort studies were included to determine subcutaneous hydration outcomes; however, reviews exploring medications also included retrospective studies and case reports. The JBI grades of recommendation were used to derive the grading score to inform the strength of recommendation for the intervention (Joanna Briggs Institute Levels of Evidence and Grades of Recommendation Working Party, 2013) [22]. Data synthesis for hydration and medications are provided as a final summary of recommendations. Grade A indicates the intervention’s desirable effects outweigh undesirable effects with adequate supporting evidence [18]. A weak recommendation (Grade B) depicts desirable effects outweigh undesirable effects, although this is not as clear or evidence not of high quality. Where there was insufficient evidence, we determined that a recommendation is inconclusive.

## Results

**Fig 1:**
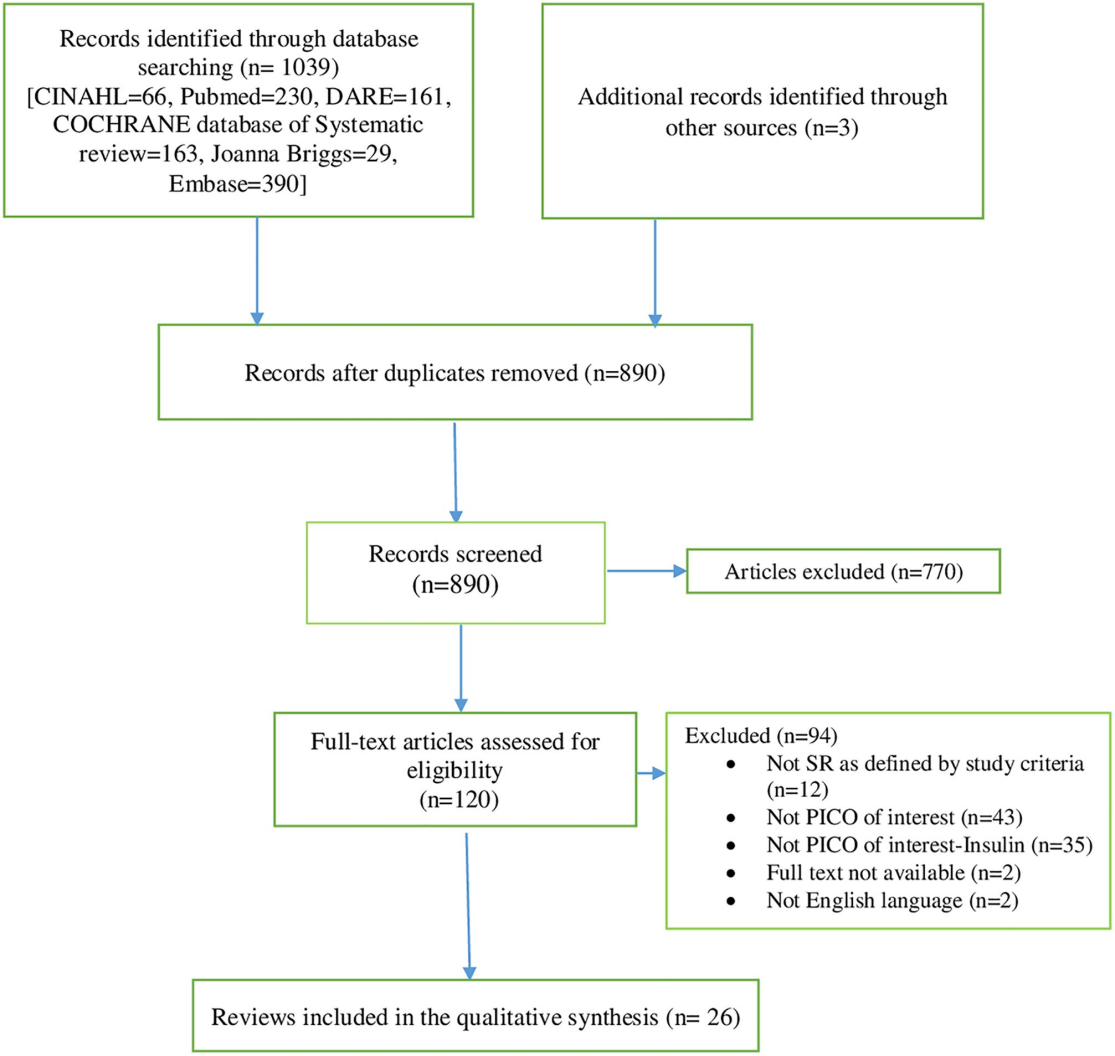
PRISMA flow chart. The diagram details our literature search and screening process for selection of systematic reviews included in the systematic review. Adapted from: Moher D, Liberati A, Tetzlaff J, Altman DG, The PRISMA Group (2009). *P*referred *R*eporting *I*tems for *S*ystematic Reviews and *M*eta-*A*nalyses: The PRISMA Statement. PLoS Med 6(7): e1000097. doi:10.1371/journal.pmed.1000097.

The search strategy yielded 1042 potential systematic reviews (Fig 1), of which 922 were excluded through abstract and duplicate screen. Of the remaining articles, 94 were excluded after full text examination against inclusion criteria in the full text screening for reasons described as above (as listed in S3 Table).

### Characteristics of included studies

Table 1 details the 26 systematic reviews of subcutaneous infusion of medications and/or fluids with 14 focused on medications only, 9 on fluids and 3 on both types of therapies. The publication dates of reviews ranged between 1997 [23] and 2019 [24, 25]. Most reviews focused on adult populations generally or specifically, for example, pregnant women and adults with amyotrophic lateral sclerosis (ALS). Two reviews, however, are specifically paediatric [26, 27]; eight all ages [15, 16, 25, 28, 29–32]; two focused on older adults and paediatrics [33, 34] and one where age was not reported [24].

**Table 1:** Characteristics of systematic reviews.

Lead author, [citation]	Aim (Intervention (Outcome)	Design and number of studies (applicable to review/total studies)	Search strategy	Population	Quality appraisal tool & rating	Author’s overall conclusion	Limitations
**SC infusion of medications**							
Abolhassani [29]	Immuno-globulin (Ig) SC versus IV: effectiveness and safety	47 studies 10 clinical trials 17 prospective cohorts 20 retrospective cohorts	Databases: 7 Date: up to January 2012	Adults (67.7%) and children with Primary Antibody Deficiencies Sample size ranges: 8–2472. Settings: NR	RCTs—criteria based on Cochrane ROB; cohort studies based on binary (yes/no) variables, using criteria for investigating the methodological quality of studies. Low quality- 15 Moderate quality– 17 High quality- 15	Improvement in patients’ quality of life and health perception when hospital-based IVIg changed to home-based SCIg (19 studies, 1028 patients). Home-based SCIg injections tolerated and preferred more than IVIg by pediatric and adult patients SCIg replacement therapy achieves acceptable IgG serum levels.	Studies may have enrolled patients who had experienced systemic AEs on IVIg thus may have increased reports of local and systemic AEs while on SCIg
Al Nofal [24]	Glucocorticoid treatments: effectiveness	34 studies; 3 included continuous hydrocortisone SC infusion 2 crossover multicenter RCTs 1 open-label clinical study	Databases: 6 Date: up to July 2016.	Patients with adrenal insufficiency. Sample size = 50 (range 7–33) Age and Settings: NR	RCTs–Cochrane ROB tool; observational studies—Newcastle Ottawa scale. Medium—high risk of bias in RCTs Fair quality for observational studies.	Continuous subcutaneous infusion of hydrocortisone may have a better effect on QoL than conventional therapy	Only 3/34 studies synthesised relevant to SCI. Low quality evidence (comparison across studies, methodological limitations, substantial heterogeneity, and moderate risk of bias) QoL data only on short-term follow-up
Barnes [26]	Prostacyclin (treprostinil): efficacy and safety	1 RCT/ 17 RCTs relevant to SCI	Databases: 3 Hand searches Dates: up to September 2018	Sample size: 470 Age: age 8–75 years Diagnosis: Pulmonary Arterial Hypertension	Cochrane ROB: low risk of selection and reporting bias;	SC treprostinal may improve cardiac function (e.g., cardiac index, right atrial pressure,) and reduce dyspnea. Although there was statistical significance in the difference in 6MWD with the use of prostacyclins, the overall effect did not meet the minimum clinically important threshold of 41 metres. Adverse events reported mostly related to the medication but also reports site irritation and redness	Short follow-up duration Meta-analysis includes all types of interventions No RCTs compared IV to SC head to head. Industry-sponsored
Bell [35]	Ketamine as an adjuvant to opioids: effectiveness and safety	3 studies relevant to SCI. 1 RCT	Databases: 3 plus 2 clinical trial registers Dates: 2012 to end Dec 2016/Beginning Jan 2017; (revised Cochrane review)	Adult patients with refractory cancer pain Sample size: 185 Age (mean): 63 years (ketamine group), 64.3 (placebo group) Setting: palliative care	Cochrane ROB tool; RCT had unclear ROB.	No reliable indication of the likely effect of ketamine, at any dose, as an adjuvant to opioids in cancer pain can be provided. Adverse events (eg hallucinations and cognitive disturbance) reported for higher doses of ketamine. Two serious adverse events reported in RCT thought to be possibly related to the study drug.	Low quality evidence is low and cannot provide a reliable indication of likely outcome effects
Bredlau [30]	Ketamine treatment: effectiveness and safety	3 studies relevant to SCI (adults) 1 RCT 1 uncontrolled trial 1 open label study 4 studies (pediatrics): 3 case reports 1 retrospective review	Databases: 2 Dates: NR	Adults and children with cancer-related pain Sample (adults): 234, range 29–181 Sample size for 3 paediatric studies: 16; range 1–11	No assessment tool reported. Discussion includes a consideration of the quality of the evidence–e.g., limitation of small number of studies with small sample sizes, few blinded RCTs and diverse methodologies.	Recommended dosages are from 0.05 to 0.5 mg/kg/h (IVI or SCI). Ketamine may be a viable option for treatment refractory cancer pain	Limited high level data available. No validated ROB tool used to assess quality of studies Only 2 databases searched
Fisher [15]	Deferiprone versus desferriox-amine (DFO): safety and effectiveness	17 RCT studies; 8 compared deferiprone alone and DFO alone; in 4 of these 8 trials reported subcutaneous infusion of DFO	Databases: 4 plus other medical databases and trial registries Dates: up to 05 March 2013	Adults and children with transfusion-dependent thalassaemia Sample size = 260 Age Range 8–49 (1 study), NR (1 study), >4 yrs with mean of 11 yrs in 2 groups and 13 in 1 group for 1 study; and >13 yrs with mean 20 and 21 years for the 1 study Setting: outpatient clinic; day care centre; paediatric hospital	Cochrane ROB tool. Unclear risk—3 Low risk—1	Deferiprone is still indicated for treating iron overload in people with thalassaemia major when DFO is contraindicated or inadequate. Intensified DFO treatment (by either subcutaneous or intravenous route) or use of other oral iron chelators, or both, remains the established treatment to reverse cardiac dysfunction due to iron overload. Adverse events are increased in patients treated with deferiprone compared with DFO and in patients treated with combined deferiprone and DFO compared with DFO alone.	Only 4 SCI trials included in review Reporting of long-term outcomes was limited and inconclusive
Fortin [25]	Medication interventions to improve adherence to iron chelation therapy: effectiveness	15 medication intervention RCT studies: 11 RCTs compared SCI of DFO with oral chelation therapy	Databases: 8 plus other medical databases and trial registries Dates: up to 01 February 2017	Children, adolescents, or their caregivers, and adults with sickle cell disease, transfusion-dependent or non-transfusion-dependent thalassaemia Sample size = 1024 Age Range 3–54 Setting: outpatient clinic; chronic care centre; multicentre clinics; pediatric hospital	Cochrane ROB tool. Unclear /low—5 Unclear/high risk– 6	Uncertain if oral chelation therapy improves adherence compared to SCI of DFO. Uncertain if oral chelation therapy reduces the risk of serious adverse effects compared to SCI of DFO. Uncertain if oral chelation therapy reduces mortality compared to SCI of DFO.	Overall quality of evidence was low to very low due to trials at serious or very serious ROB, outcome estimates imprecise, restrictive exclusion criteria, use of non-validated QoL measurements and lack of reporting of data.
Gaudet [63]	SCI of terbutaline by pump for tocolysis: safety and effectiveness	15 studies: 2 RCT 1 nonrandomized trial 2 prospective cohort 7 retrospective cohort 2 case series	Databases: 5 Dates: 1950–2011	Pregnant women with preterm contractions. Sample size: NR; range = 9–1366 (average 291 ± 395) Settings: single center sites and a national outpatient perinatal program database	Confounding, selection, performance, detection and attrition bias assessed; selected items from the McMaster Quality Assessment Scale of Harms; overall risk of bias ratings: Low—1 Medium—7 High– 7	Use of pump therapy should be limited to the research setting Concerns regarding safety of therapy persist.	Confidence in the validity and reproducibility of evidence is low
Lingman-Framme [31]	SC immunoglobulin versus IV: effectiveness, safety, efficiency	25 studies 2 RCT 18 observational studies 5 health economic studies	Databases: 9 Dates: NR	Patients with primary or secondary immune-deficiency Sample size: NR; range = 8–83 (from few studies that reported) Age: any age Setting: NR	GRADE, Swedish Council on Health Technology Assessment 2 RCT–moderate 9 observational -moderate; 9 observational–low; 5 health economic–low	Little evidence of difference in AEs between IV Ig and SC Ig substitution Comparisons between SC and IV administration difficult as two methods used to calculate the SC substitution dose. No firm conclusion as to whether it was home treatment per se that improved HQOL with SCIg. Estimation of costs in the older studies is outdated.	Quality of evidence low with diverse methods of measuring and reporting infections Major differences between national health and insurance systems of countries and different price policies of the products in different countries complicate the generalization of results
Orrell [36]	SC anti-oxidant treatment vs placebo: effectiveness	9 studies; 1 relevant to SCI RCTs or quasi-experimental studies	Databases: 3 Dates: 1966–2005	Adults with amyotrophic lateral sclerosis Sample size: 110 Age: mean 58 years Setting: NR	Cochrane ROB approach; Unclear risk concealment and blinding Adequate risk diagnostic criteria, outcome, baseline differences and completeness of follow up	No benefits of acetylcysteine in survival at 12 months No significant evidence of beneficial effect in people with ALS.	Meta-analysis includes all types of interventions Studies were poorly designed, and underpowered, with low numbers of participants and of short duration
Paramothayan [37]	Prostacyclin or one of its analogues in: effectiveness	9 RCTs; 2 studied treprostinil vs Placebo.	Databases: 3 +; Drug companies were contacted for relevant trial data (published and unpublished)	Adults with idiopathic primary pulmonary hypertension (IPPH) Sample Size: 496 Mean age 37 & 44.5 Settings: multi-centre but specifics NR	Cochrane approach to concealment of allocation. Additional assessments used the Jadad 5-point scale	Although some of the side effects described are tolerated reasonably well by patients, potentially serious and life-threatening complications can occur, mainly related to the drug delivery system with intravenous administration. SC Treprostinil may have an important role in treating patients with moderate symptoms who are not severe enough to require intravenous prostacyclin.	Studies were performed over a short period of time (eight—12 weeks), thus limited data on survival or side effects over a longer period
Reichmann [62]	SC metoclo-pramide and ondansetron to treat nausea and vomiting: effectiveness	5 studies 1 retrospective non-randomized matched cohort study, 4 retrospective case series	Database: 1 + Google scholar. Dates: No date or language restrictions	Pregnant women with nausea and vomiting Sample size: 2251 Range: 301–876 Setting: NR	Level of evidence reported: LeveI II—1 Level III—4	Due to low quality study designs, SCI should be limited to intractable hyperemesis unresponsive to more-conventional treatment	Limited quality evidence All studies industry-sponsored and -authored non-randomised reports.
Schmidt-Hansen [32]	Buprenorphine: effectiveness and safety	19 RCTs; 1 relevant to SCI	Databases: 5 + other resources Dates: 1948–2015	Adults and children with cancer pain	Cochrane ROB Unclear to high risk for 11/12 criteria	Sublingual buprenorphine faster onset of pain relief compared to subdermal buprenorphine Similar duration of pain relief No significant differences in AEs	Low quality evidence Difficult to say where buprenorphine fits in the treatment of cancer pain with strong opioids.
Stoner [38]	Medication administration (e.g., PCA) SC vs IV: acceptability, safety.	6 studies; 3 RCTs relevant to SCI	Databases: NR Dates: 1974–2014	Adults Sample size = 39 Age: mean 45, range 18–85 Setting: hospital; elective abdominal or extremity surgery	Cochrane ROB e—1 Low-2	4/6 studies—patient preference for SC over IV because SC at home and time saving Patient preferred treatment will influence adherence and patient experience/satisfaction 1 study showed comparable efficacy and safety profiles (IV SC PCA)	Unclear if other studies are SC injections or SCI
**SC infusion of fluids/ Hypodermoclysis (HDC)**							
Forbat [42]	SCI: mechanisms, locations, duration, quantity, and type	14 studies 4 systematic review 2 RCT 1 controlled trial 3 prospective 1 survey 1 descriptive 2 retrospective comparative	5 databases Dates: all	Advanced illness population Sample size 12–290, 1 postal survey of doctors n = 1054 Age: > 64 Settings: palliative, LTC, older community setting	Critical Appraisal Skills Programme Low—4 Medium—4 High– 6	Studies under-report the mechanism (site, mode, frequency and volume) by which artificial hydration is provided, creating a paucity of evidence-based guidance by which to practice.	No robust evidence, particularly in non-malignant populations to inform practice
Good [49]	Medically assisted hydration: QoL and length of life.	4 studies relevant to SCI 3 RCT 1 prospective comparative study (PCS)	Databases: 8 Dates: 1966-March 2014	Adult patients with advanced cancer Sample size = 533 Age: NR Setting: palliative care	Oxford Quality Scale for RCTs + for this update Cochrane ROB tool; Rinck scale for PCS. Low risk: 2 RCT High/unclear risk: 1 High risk:1 PCS	No significant benefit in the use of medically assisted hydration in palliative care patients	Limited evidence to inform definitive recommendations
Ker [33]	Insertion of different parenteral access methods: reliability, ease of use and speed of insertion	17 studies; 11 relevant RCTs	Databases: 6 & reference lists and Google Dates: Not limited	Older adults/ patients with diabetes and paediatrics Age: 10 RCTS–Adults >65 years Paediatric 1 mth—10 years with mean ~2 yrs.	Cochrane ROB All RCTs had mostly unclear/ high risk of bias	Sufficient evidence to conclude that peripheral intravenous access is best if achieved easily for larger volumes of fluid compared to than other routes; but if this is not possible, the intraosseous and subcutaneous routes are viable alternatives Subcutaneous route may be suitable for patients who are not severely dehydrated but when ongoing fluid losses cannot be met by oral intake	Limited quality of evidence due to lack of adequately powered trials at unclear/high risk of bias.
Marikar [27]	SCI vs IVI for moderate dehydration	3 studies 1 RCT 1 case series 1 multicentre pilot	Database: 3 + plus other medical and trials registries Dates: NR	Paediatric Sample size = 235 Settings: Hospital, emergency department	CEBM levels: 2B:1 (individual cohort including low quality RCT?) 4: 2 (case series and poor-quality cohort and case-control studies?)	1 industry-sponsored RCT suggest that in the ED setting, SC fluids are non-inferior to IVI in cases of mild to moderate dehydration with quicker mean times for line placement and greater parent/caregiver satisfaction. Most patients experienced infusion-site reactions which improved without further intervention; no serious adverse events. 2 studies reported successful rehydration with rHFSC fluids alone	Limited safety data are available No studies compare SC fluids alone against IV; Existing trials have potential bias-sponsored by a pharmaceutical company producing rHf
Remington [46]	SCI vs IVI for mild-moderate dehydration	8 studies: 2 RCTs 3 prospective cohort 1 prospective observational 2 retrospective chart review	Databases: 5 Dates: 1996–2006	Older adults Sample size: 508 Age: 71–85 Setting: hospital, acute geriatric units, LTC, hospice	No assessment tool reported Discussion includes a consideration of the quality of the evidence (e.g. small sample size, use of non-standardized evaluation methods)	HDC effective as IV rehydration of older adults with mild to moderate dehydration Advantages of HDC include the same number of or fewer complications, cost savings, greater patient comfort, and less nursing time to start and maintain the infusion Remains unclear why HDC is used infrequently in the US.	Methodologically rigorous research remains scarce
Rochon [23]	SC hydration: safety, efficacy	12 studies 2 RCTs 10 case reports/series	Database: 1 Dates: 1966–1996	Adults with dehydration Sample size: 668 Age: range 32.5–91.3 Setting: NR	No assessment tool reported Discussion includes a consideration of the quality of the evidence as poor	HDC with electrolyte-containing fluids is safe Hyaluronidase to promote absorption remains unresolved Limited evidence suggests potassium chloride may, with caution, be safely added to SCI HDC may be ideally suited for the treatment of dehydration as metabolic imbalances do not have to be corrected immediately Fluid is absorbed similarly using either route (based on 2 RCTs). Avoid electrolyte-free solutions- adverse effects HDC is an option for treating dehydration when patients have inadequate oral intake	Majority of available studies are of poor quality
Rouhani [28]	Oral vs IO, NG, IP, SC, rectal for mild to moderate dehydration: safety, effectiveness and efficiency	3 studies on HDC: 2 case series 1 case report	Databases: 5 Dates: up to 2009	Paediatric Sample size: 56 Age: <18 years Setting: emergency department	No assessment tool reported Discussion includes a consideration of the quality of the evidence as poor (e.g. small sample size)	Only 1 case series showed benefit of HDC; more research is required before its use in pediatrics can be endorsed HDC may represent life-saving alternatives if IV access cannot be obtained, particularly in resource-limited settings, where limited supplies necessitate flexible treatment plans Oral rehydration remains the preferred route for dehydration	Additional evidence is needed before HDC can be endorsed.
Turner [47]	5% dextrose SC vs IV: safety and effectiveness	4 studies 2 RCTs 1 cohort 1 systematic review	Databases: 9 Dates: 1993–2003	Adults Sample size: 211 Age: Mean 53 and 80 years (2 studies) Setting: LTC, hospital	No assessment tool reported Discussion includes a consideration of the quality of the evidence as poor (e.g. outcomes not reported separately, no blinding)	Appropriate volumes of SCI of dextrose (in the form of half-normal saline-glucose 5%, 40 g/L dextrose and 30 mmol/L NaCl, or 5% dextrose solution and 4 g/L NaCl, or two-thirds 5% glucose and one-third normal saline) can be used effectively for the treatment of dehydration, with similar rates of adverse effects	Evidence is limited, and larger randomised controlled trials using validated outcome measures needed
Wilhelm [34]	Hyaluronidase-facilitated SC fluids in pediatrics and older adults and low resource settings: effectiveness and safety	7 studies: 3 RCTs 1 phase IV single arm 1 prospective observational 1 observational 1 retrospective	Databases: 1 Dates: NR	Pediatric and Older Adult populations Sample size: 513 Age: pediatric Old adults >80 years Settings: emergency departments, acute geriatric units, long-term care	JADAD score/ Oxford quality scoring system/Eggers scale for bias but not reported.	Enzyme-assisted Subcutaneous Fluid Administration (EASFA) is comparable to intravenous fluids for infusion rates in both pediatric and older adults with mild to moderate dehydration. Higher rates of insertion success with subcutaneous catheters proves it to be more useful in some situations.	Quality of studies not reported
**SI infusion of fluids and medications**							
Duems-Noriega [16]	Drugs and fluids SC vs IV, IM, oral, placebo: safety, effectiveness, acceptability and procedure	178 studies included (letter, review and communication); 103 studies reported: 49 RCTs 1 uncontrolled 2 prospective 1 prospective cohort 4 non-RCTs 2 retrospective 20 case series 20 case reports 1 survey 3 open trial	Databases: 4 Dates: NR	All ages and palliative population Sample size: range 1–235 (drug administration); 12–150 (hydration care) Setting: LTC, hospital, health care centres	SIGN A: 3 B: 48 C: 43	SCI is an effective alternative for rehydration in patients with mild-moderate dehydration Drugs administered by SCI are well tolerated and associated with minimal side-effects but may be absorbed slower. Factors influencing absorption/ tolerance: hypovolemia, oedematous, state or shock, hypoalbuminemia, hypotension, connective tissue at site, decreased site perfusion, shallow injection depth, large molecule drugs, non0neutral pH drugs (buffers such as phosphate, carbonate, citrate or histidine may be used), hypertonic diluents, liposoluble irritants, high viscosity, local anaesthetic or cold which causes vasoconstriction). Hyaluronidase increases local absorption.	
Fonzo-Christe [48]	SC rehydration in the elderly	4/25 Drug Studieswere SCI: 3 RCTs 1 pharmaco-kinetic trial 6/25 Fluid Studies: 1 systematic review 4 RCTs 1 prospective cohort	Databases: 2 Medline (1966—March 2003) and Embase Geriatrics and Gerontology (1992–2002)	Sample size (drug studies): 178; range 8–120 Age: range 22–83 yrs Sample size (fluid studies): 222 & 685 for systematic review; range 6–85. Age: range 52–100 yrs Settings noted: surgical, healthy volunteers, rehabilitation	Adapted French guidelines of ANAES (level of evidence graded by study design)	34 drugs reviewed, only 13 (38%) licensed for subcutaneous use in Switzerland, UK, France or Germany, although drugs are used frequently off label Haloperidol and furosemide are used off-label and there are no well-designed studies supporting their subcutaneous use. SCI is effective and safer (fewer reactions at catheter site), treatment of choice for confused patients with smaller metabolic and hormonal changes.	Only morphine (14 articles of 68) and rehydration (six articles) are evaluated in high level studies. Only fentanyl, hydromorphone, pethidine, butylscopolamine, ceftriaxone and chlorpromazine studied in at least 1 well-designed study No levels of evidence given for individual studies
Gomes [39]	SC drugs and fluids: nurses knowledge	9 studies 3 literature reviews 1 case study 5 quantitative	Databases: 5 Dates: open period of time	Adults Sample size: 1 to 79	Level of evidence reported: Level 1: 1 Level VI: 4 3 did not fit classification	SCI remains underused, and there is an urgent need for more clinical studies to promote decision-making and to guide clinical practice among professionals. HDC is considered a simple, safe, and effective practice for hydroelectrolytic replacement and/or drug therapy	Small number of studies with small sizes limited reporting—also low evidence studies.

AE–adverse event; CEBM—Centre of Evidenced Based Medicine; HDC–hypodermoclysis IO–intraosseous; IM–intramuscular; IP–intraperitoneal; IV–intravenous; IVI–intravenous infusion; LTC–long term care; NG–nasogastric; NR–not reported; O–Oral; QoL–quality of life; rHFSC–recombinant human hyaluronidase-facilitated subcutaneous; ROB–risk of bias; SC–subcutaneous; SCI–subcutaneous infusion; SIGN–The Scottish Intercollegiate Guidelines Network

The systematic reviews incorporated a diversity of study designs and quality levels from case studies to systematic reviews and randomised controlled trials. However, only 9 reviews included only randomised controlled trials [15, 32, 33, 35–38]. Similarly, sample sizes varied depending on the number of SCI studies included in the review synthesise of findings with very few meta-analyses performed due to varied study designs, outcomes and size.

### Methodological quality

The AMSTAR 2 quality scores of the included reviews are described in S4 Table. While 10 reviews achieved an overall confidence rating of high, sixteen of the reviews (61.5%) had critical methodological weaknesses (3 studies with moderate, 5 low and 8 studies rated as critically low). The main deficiencies noted were failure to report a priori protocol, study design selection, full details of excluded studies, funding sources of primary studies and results reflecting risk of bias assessment. The Cochrane risk of bias tool or an adaptation was the most commonly used tool by systematic review investigators to determine quality of the primary RCT studies (*n* = 12/26), while 5 only provided a narrative discussion of quality. Most review authors noted that results should be interpreted with caution due to study limitations.

### Mechanisms of subcutaneous access

Limited data were reported on the materials and techniques used to achieve subcutaneous access for hydration or medication therapy. Table 2 describes the characteristics of SC device design and dwell-time, subcutaneous anatomical sites and insertion techniques and infusion delivery systems in relation to studies reporting on hydration therapy. No studies compared the safety and efficacy of the various characteristics, including mode of delivery. The findings for hydration and medication SCI are presented separately.

**Table 2:** Subcutaneous access and infusion delivery device protocols.

Catheter Design and Dwell Time	Subcutaneous Sites	Insertion Technique	Infusion Device
**Gauge:** 19 [16]; 21–27 G [16]; 23-27G [39]; 25 G [16,27,42] is less likely to reach muscle; gauge is dependent on patient condition and type of solution[39] **Access device**: butterfly needle [16, 38]; butterfly replaced by silastic needle/IV cannula to manage irritation [42]; IV cannula 24G or butterfly 25G (1 study IV cannula ↑ dwell time but insertion more difficult than butterfly) [39] **Site change**: 2 d [16, 26, 27, 42]; up to 3 d or longer [16]; 3 d [42]; 7 d [42]; morphine and hydromorphone 2 or 5 d [39]; mean 11d with IV cannula vs 5 days (3-7d range) with butterfly [39]	**Minimum skin thickness**: 1–2.5 cm [16] **Most common:** abdominal wall^a^ [39]; upper chest (infra-clavicular,^b^ [39] **Other:** intrascapular^c^ [16, 39, 42]; upper extremities (deltoid region preferred) [16, 39, 42]; flank [26]; hips [26]; thighs^d^ [39, 42] **Contraindicated**: bony prominences [16]; joints [16]; previous surgical incision [16]; radiotherapy [16]; damaged skin [16]; intercostal space in cachectic patients (high risk of pneumothorax) [39]; near mastectomy, tumour, ascites, lymphedema [39]; inner thigh if urinary catheter [39]; thigh if peripheral vascular insufficiency [39]	Cleanse skin with povidone-iodine or chlorhexidine 2%. Flush cannula with at least 0.2 mL diluent. Pinch skin to pull subcutaneous tissue away from skin. Insert needle at 45–60 degrees. Aspirate to rule out venipuncture. Apply dressing [16]	**Hydration:** pump [27,42, 48]; gravity ^e^ [13, 42, 48, 50] **SCIG**: syringe driver pump, pump/ portable pump [16, 27, 38, 48] **Medications:** syringe driver pump [39] **Furosemide:** pump [16], elastomeric [16] **Terbutaline:** pump [39]

G–gauge; d–days;

^a^ abdominal wall useful for hydration, with left iliac fossa considered the preferred zone (with maximal distance between colon and abdominal wall[16]; consider a circumference around the navel with about 4 fingers around it[26];

^b^ lower distribution surface of chest is useful for drug delivery; chest region for men and inframmary region for women;

^c^ suprascapular and interscapular areas provide large distribution surface and beneficial in patients with confusional or agitated states, or tend to pull IV out (due to difficulty of access for patients and do not interfere with patient’s mobility)[16, 26];

^d^lower limbs (lateral aspects of thighs) are most painful places with lower distribution surface limiting administration[16];

^e^clinicians did not alter the gravity flow rate, allowing it to freely adjust to its own speed as permitted by gravity and the tissue;

### Subcutaneous hydration

S5 Table describes the indications and contraindications for SCI for hydration, as described in the studies. The most common indicator was mild to moderate dehydration, with the most common contraindication being rapid/ high fluid volume requirements.

### Population

Subcutaneous hydration has been primarily studied in the hospital sector (n = 6), palliative care and hospice setting (n = 3), long term care (n = 1) and one study discussed the home care setting as being appropriate (however, no data provided) [39]. One RCT, one multicentre pilot, 2 case series and 1 case report studied the pediatric population.

### Infusion properties

Table 3 presents the reported and extrapolated properties of the hydration solutions. In the elderly population, the mean daily volume was 1340 mL (range 698–1708 mL) or a bolus of 500 mL over 2–6 hour) for a mean total of 5 days (.25–21 days). In the pediatric population, a mean volume of 365 mL of hyaluronidase-facilitated isotonic solution was infused for a mean 3.1 hours. For terminally ill patients, the mean daily volume was 1068 mL (range 698–1708 mL). One study, however, suggested that results may not show benefit for SC hydration in palliative care as the typical 1000 mL volume used may be insufficient and that the optimal volume is undetermined [40]. Interestingly, another study of 100 terminally ill patients concluded that SC is useful and safe, with a maximum 1708 mL/24 h [41]. However, Forbat et al. [42] concluded from their systematic review of 14 studies investigating subcutaneous hydration at end of life that there is a lack of empirical evidence to guide use of this therapy.

**Table 3:** Subcutaneous hydration infusion properties (analytic studies).

Study	Population	Infusion		
Design [Quality*] (*Review study*; primary study)	Size Type Setting	Solution + Additives	Volume Rate	Frequency Duration
A. RCT [NR] [27,33,34,50]	*n* = 148 Children Hospitals	Isotonic + hyaluronidase^	Mean 365 mL 31.2 ml/kg x 1 hour (Mean 15.4 mL/kg/hr)	Mean 3.1 hours
B. RCT [High] [33,34,46–48,53]	*n* = 96 Older adult Hospital	0.45% NaCl +D5W	750 mL/day (Range 457–1500) 500 mL bolus x 2–6 hours	NR Median 6 days (1-36d)
C. RCT [NR] [33,34,54]	*n* = 67 Older adult Hospital	0.9% NaCl D5W+0.45% NaCl (+ up to 20 mEq KCl/L prn	1320 mL ± 400 mL Max 1.5L/day/route	NR 72 hours
D. RCT [High] [16,23,42,33,34,46–48, 44]	*n* = 60 Older adult Hospital	0.45% or 0.9% NaCl, D5W (+ up to 10 mmol/L KCl prn)	Max. 2L/24 hours (continuous) Mean 3.3 L over 48 hours	NR At least 48 hours
E. RCT [NR] [16,55]	*n* = 57 Older adult Hospital	0.45%/0.9% NaCl + dextrose 2.5%	Max. 1.5 L/24 hours	NR
F. RCT [NR] [16, 23, 33,52]	*n* = 34 Older adult Stroke	40 g/L D + 30 mmol/L NaCl ^	2L/24hours	NR 3 days
G. RCT [High] [23,33,48,56]	*n* = 6 Older adult Hospital	D5W +4g/L NaCl	1000 mL 167 mL/hr	6 hours
H. Prospective [NR] [16,46,57]	*n* = 57 NR	NR	1161 mL/24 hours NR	NR 15.9 days
I. Prospective [Mod.-High] [16,34,46–48,44]	*n* = 55 Older adult Dementia Long term care	0.9% NaCl, 67% D + 33% NaCl	NR 5–75 mL/hr	Daily (49) / As required (6) 21 days (maintenance hydration); 11days (acute dehydration)
**PALLIATIVE**				
J. RCT [Moderate-High] [42,49,40]	*n =* 129 Cancer Hospices	NaCl Placebo NaCl 100 mL	1000 mL 250 mL/hr x 4 hours	NR
K. RCT [NR] [47,58]	*n* = 42 Advanced cancer	D5W +140 mEq NaCl	1000 mL 42 mL/hr	NR 48 hours
L. Prospective [High] [41,42]	*n* = 100 Palliative care unit	0.9% NaCl or 2/3 +1/3 + 750 units hyaluronidase/L	Mean 1203 ± 505 mL Mean 72 ±18 mL/hr	17±6 hours/day Avg. 14-18d

*–Quality as reported by systematic review authors;

^– 1500 units hyaluronidase added to each bag if infusion ran behind time; Avg–average; D–dextrose (glucose); D5W—5% dextrose solution; hr–hour; LTC–long term care; Max.–maximum; Mod.–moderate quality; NaCl–sodium chloride; NR–not reported

Typical solutions in the elderly and palliative population were either sodium chloride (0.45% or 0.9%) or, more commonly, a dextrose/saline solution. Hyperosmolar, colloidal or hypertonic solutions without electrolytes are not recommended [16, 23]. Potassium chloride in amounts considered to be maintenance fluids (10 mEq/L and 20 mEq/L) was added to the solution on an as-needed basis in 2 studies [16, 43]. No studies compared the safety or efficacy of these specific hydration solutions. There is insufficient evidence to recommend Ringer lactate solutions [16]. Lastly, although many of the included studies had a standard infusion protocol (e.g., rate and volume), O’Keefe and Lavan [43] recommended the need to tailor fluid therapy to individual patient requirements, using frequent biochemical measurements.

Infusion rates varied. Rochon et al. [23] acknowledge the wide range of reported hydration rates (75–1250 mL/hour), however, due to reports of adverse events at high rates, they recommended a rate of 50 mL/hr to treat dehydration in older people. In the largest RCT of 34 patients, in which there were 2 reports each of erythema and hyponatremia, the rate of infusion was 83 mL/hr of a dextrose-saline solution. In the 5 cases of serious adverse events, rates were reported between 480 mL—675 mL/hr of either 5% glucose or dextrose combined with Amigen and 10% invert sugar (hypertonic) solutions. Duems-Noriega and Arino-Blasco [16] recommend 30–80 mL/hr or up to 1.2 L per 24 hours in one site or up to 2.4 L/day by 2 different sites, with hyaluronidase-facilitated infusion rates up to 300 mL/h.

In a review of subcutaneous hydration at end of life, rates ranged from 20 mL/hour to 2400 mL/day, with the most commonly reported rate of ~75 mL/hour [42]. In one of the included palliative care study, 48% reported continuous overnight infusions at 60–120 mL/hour, and 21% received a bolus of 500 mL over one hour, one to three times per day. Five studies reported overnight infusions, in the elderly and palliative care population [42–44]. An RCT of 21 patients with cancer found many patients prefer bolus infusions of 500 mL twice daily as compared to overnight (p = 0.0013 for patients, and p = 0.062 for investigators), while investigators either preferred bolus infusions or found no difference [45]. The authors caution that 14% of those receiving boluses withdrew from the study due to swelling. However, all four withdrawn patients had infusion sites near the groin (2 males) and near the breast (2 females).

Duration of treatment ranged from a mean of four to 21 days for SC hydration in 1 review with an average of 10.5 days [46], and as long as three to six months in another review [16]. In the palliative population, durations ranged from three weeks to six months, or one to three days [42].

### Effectiveness, safety, acceptability and efficiency: Summary

Table 4 provides a summary of the outcomes of the findings from the RCTs and prospective cohort studies included in the systematic reviews of subcutaneous hydration infusions (as detailed in S6 Table). Recommendations informed by the level of evidence are also provided in Table 4 for each of the outcomes. Overall, there is strong evidence to recommend SCI for hydration in older adults [16, 23, 34, 39, 46–48], weak evidence for pediatrics [27, 28, 33], and inclusive evidence for palliative patients [16, 39, 42, 49].

**Table 4:** Subcutaneous hydration: Summary of findings^1^.

	Older Adults (8 primary studies)			Pediatric patients (1 primary study)			Palliative patients (3 primary studies)		
	Favours IV	No difference	Favours SC	Favours IV	No difference	Favours SC	Favours no Tx	No difference	Favours SC
**Effectiveness**									
Biochemical restoration		++++++	+						
Clinical improvement		+++++	+		+				
Volume infused	+	+++		+	+	+			
Duration of infusions		++			+				
Hydration		+++						+	
Weight changes					+				
Insertion success		+			+				
Survival								+	
GRADE	**A**			**Inconclusive**			**Inconclusive**		
**Safety**									
Local adverse event		+++++++			+			+	
Site pain/procedure discomfort		+			+				
Hyponatremia		++							
Cardiac failure		+							
Fluid overload		+	+						
Catheter dislodgment		++	+						
Agitation/delirium			+					+	
Nausea, thirst									+
	A			**B**			**Inconclusive**		
**Acceptability**									
Patient/caregiver satisfaction						**+**			
Activities of daily living		+							
Difficulty inserting catheter						+			
Perception of feasibility (ease)		+	+			+			
Quality of life									++
GRADE	A			**B**			**Inconclusive**		
**Efficiency**									
Insertion times		+						+	
Total treatment time						+			
Cost of cannulae		++++							
GRADE	A			**B**			**Inconclusive**		
Overall Recommendation								+++	
**OVERALL GRADE**	A (adults)			**B** (pediatrics)			**Inconclusive** (palliative)		

^1^ Findings further described in S6 Table;

+ number of times outcomes reported within primary studies; +, moderate-high quality study;

^2^ Most were mild to moderate in severity (except 1 severe pain) and local site reactions;

Grade A: strong evidence of adequate quality describing desirable effects outweighing undesirable effects and values, preferences and patient experience supports its use; Grade B: weak evidence, although not of high quality, support its use, with desirable effects appearing to outweigh undesirable effects, although this is not as clear; Grade Inconclusive: insufficient evidence to make a recommendation in less than 50% of studies (adapted from Aromataris et al., [18]).

#### Effectiveness

Study findings of the RCTs and prospective cohort studies support the recommendation that subcutaneous hydration is at least as effective as IV therapy and may be superior in certain circumstances for the treatment of dehydration in the pediatric and elderly population (Table 4 and S6 Table. Both biochemical (e.g., urea and creatinine) and clinical improvement (e.g., general wellbeing and improvement in mentation) were reported in all studies investigating this outcome, in both SC and IV groups. One small prospective cohort study reported superiority in the SC group in urea (p = 0.001), creatinine (p < 0.001) and sodium (p < 0.05), with an overall clinical improvement rate of 77%. The only study showing a preference for IV therapy in 1 outcome measuring volume infused was in the pediatric population, in which patients who received SC hydration in the emergency department had to be switched to IV upon admission to inpatient units due to lack of staff ability to perform SC hydration, which led to reports of “0 ml” infused [50]. The study did demonstrate a statistically significant higher volume of infusion with subcutaneous infusion in the emergency department.

In the elderly population, improvement was similar to IV hydration, with two systematic reviews providing evidence that SC hydration may be the treatment of choice for patients with cognitive impairment [43, 47, 48]. Rationale cited include the ability to place catheters in discreet sites out of sight, relatively pain-insensitive sites, (although studies site comparable discomfort), ease of placement and indirect savings from not having to observe the patient as closely [43].

In the pediatric study, there was a 20% placement failure rate of children randomized to IV therapy vs no SC access failures [50]. A meta-analysis supports this finding, reporting that children and adult patients (n = 238) receiving IV hydration were more likely to experience an insertion failure (RR 14.79, 95% CI 2.87 to 76.08; GRADE rating: moderate) [33]. A systematic review concluded that the odds of correct initial needle placement was 7.19 times (2.93, 17.68), p = .04) higher in hyaluronidase-facilitated subcutaneous access than IV [34].

Both hydration and medication reviews included the use of hyaluronidase, an enzyme that facilitates subcutaneous absorption [15, 23, 27, 28, 33, 34, 39, 40, 42, 48–50]. Spandorfer and colleagues [50] report that hyaluronidase is safe, effective and well tolerated in subcutaneous hydration solutions for pediatrics. They found that gravity-driven infusion rates can be increased four-fold with better systemic fluid absorption. In the palliative care population, 150 units of hyaluronidase was injected as a bolus into the infusion site immediately before starting each twice daily 500 mL bolus over one hour of two-thirds dextrose and 1/3 normal saline [50]. No significant differences were observed for pain, swelling, edema, rash or leakage between placebo and hyaluronidase, with the authors concluding that hyaluronidase is not necessary for routine boluses, unless the patient does not tolerate the infusion well due to swelling or pain. Administration protocol varied across studies. In one study 150–300 units/L were added to the solution for continuous infusions; however, for hydration boluses, 150 units was injected into the SC site before the infusion [51]. A protocol for pediatrics was to insert the SC set and then flush it with 140/150 units hyaluronidase prior to starting the infusion and repeating the dose once every 24 hours during infusion, as needed [27, 34]. Other protocols included using hyaluronidase only if the infusion was running slowly (method not reported) [52] or if problems with resorption as a bolus injection before starting the infusion [53].

#### Safety

Evidence suggests that subcutaneous hydration in the pediatric and elderly population is safe and equivalent to IV hydration, in terms of local site reactions and systemic events and superior in catheter extraction and device-related agitation (see Table 4).

In the pediatric population, most patients in both SC (100%) and IV (90%) experienced at least one adverse event (no serious events) [33]. There were fewer case of erythema (RR 0.43, 95% CI 0.31 to 0.61) and edema at the insertion site (RR 0.42, 95% CI 0.25 to 0.72; n = 453; p = 0.001). The majority were local site reactions (swelling, erythema and pain), which Spandorfer et al. [45] reported were resolved spontaneously without treatment. These authors also reported the pain as being related to needle insertion in both groups with resolution post-insertion. Duems Noriega and Arino-Blasco [16] report that these local reactions are frequent but easily avoidable (through rate and volume control, proper needle placement [avoiding muscle] and aseptic technique). Ker et al.’s meta-analysis [33] found no evidence that the number of patients reporting pain differed between IV and SC (RR 1.01, 95% CI 0.83 to 1.22; n = 262; p = 0.94). More patients experienced phlebitis with an IV device than SC device (RR 5.04, 95% CI 1.14 to 22.30; n = 181). Infection (e.g. cellulitis and lymphangitis) rates were higher in patients with IVs than SCI (RR 3.70, 95% CI 1.06 to 12.88; n = 211; p = 0.04) [33].

The most common, although rare, systemic reactions reported to be possibly related to SC therapy include hyponatremia in a total of 3 patients, cardiac failure in 2 patients, and fluid overload (mean 0.003 episodes per day of therapy), all of which were comparable rates to IV [46, 44, 52, 53].

Catheter dislodgments, in which the catheter/needle is pulled out, were more likely to occur with IV than SC in the elderly population (RR 3.78, 95% CI 1.16 to 12.34, p = .03) [34]. Placement of the SC catheter in this population in discreet locations, such as the upper back (which is out of sight and less accessible) leads to less dislodgements [43].

#### Acceptability

Subcutaneous hydration is significantly favoured over IV hydration with regards to successful and ease of device placement, patient agitation, and patient/caregiver satisfaction (Table 4). Patients with an IV are more likely to be agitated than with SC [33, 43].

In an RCT of 96 elderly patients, 17 patients were switched to SC (from IV) due to lack of venous access (n = 8), removal of cannulae by patients (n = 5), or other causes not reported [47, 53]. The need for medications and lack of medications suitable for SC access is an impediment, as 11 patients had to be switched to IV access to accommodate medication administration thus hydration was also converted. One study showed no nursing preference for SC vs IV; however, it is noted that the study protocol was for physician placement of IV catheters (with the latter reporting SCI as significantly more feasible [54].

Ker and colleagues [33] suggest that the ease of administration and cost-effectiveness as being particularly useful for healthcare workers with minimal medical training. In a multi-centre pediatric study, most participating centers did not have protocol or training for inpatient staff [50]. Therefore, upon transfer from the emergency department to the inpatient unit, patients had to be switched to IV therapy, which led to designation of SCI treatment failures. This finding was echoed by a qualitative study which found that 71% of nurses were unaware of this technique and 100% reported not having received any organizational guidance [39].

#### Efficiency

Subcutaneous hydration is significant favoured over IV hydration in terms of cost effectiveness, in a few measured outcomes (Table 4). Total treatment time (from first catheter insertion attempt to end of infusion) was reported in a pediatric study as significantly shorter than the IV route [50]. Two studies reported cost savings with SCI route, due to fewer and less costly cannulae required, although these did not reach statistical significance [43, 52]. Indirect costs are compounded by the additional supervision required for IV infusions [46]. Cost of IV therapy has been reported to be four times higher than SC therapy [39, 46].

### Subcutaneous medications

Ideal medications for subcutaneous administration are those that are hydro-soluble, and have neutral pH, low viscosity and low molecular weight [16]. Irritating additives should be avoided, including propylene glycol, glycerin and ethanol as these can be associated with increased reactions and discomfort [16]. Studies were not detailed for many medications, but multiple reports are included in literature supporting the use of numerous opioids. Subcutaneous infusion when compared to IV can be similar or even superior due to less reported side effects. Table 5 provides a summary of the outcomes of the findings from studies and recommendation grades informed by the strength of evidence. Medication study data was either not separated by ages or not reported in many of the reviews.

**Table 5:** Summary of findings: Subcutaneous medication.

Grade	Medication (n = number of studies)	Age	Comment
			**Antimicrobials**
**Grade A (strong evidence)**	Ceftriaxone [16,48] (n = 5)	NR, A	Lidocaine allows for greater comfort during administration. Lower Cmax^1^ and slower to Tmax^2^
	Ertapenem^a^ [16] (n = 2)	NR	Time-dependent kill gives potential benefit to use of SC administration with increase in Tmax
**Grade B (weak evidence)**	Fosfomycin [16] (n = 1)	NR	High concentrations not tolerated well
	Teicoplanin [16] (n = 1)	NR	High risk of resistance with the medication via any route so reserve use
**NOT Recommended**	Amikacin [16] (n = 2)	NR	Delayed Tmax and lower Cmax reduces bactericidal power unless used as synergy with beta lactam
	Gentamicin [16] (n = 4)	NR	Serious cutaneous reactions
	Netilmicin [16] (n = 1)	NR	Serious cutaneous toxicities
**Inconclusive**	Ampicillin* [16] (n = 1)	NR	Diluted and given over 20 minutes
	Cefepime*[16] (n = 1)	NR	
	Tobramycin* [16] (n = 1)	NR	Local minor effects may limit use although favorable clinical results
			**Chemotherapy**
**Grade B (weak evidence)**	Azacitibine [16] (n = 1)	NR	Local reactions reduced with warm compresses
	Bleomycin [16] (n = 1)	NR	SC continuous infusion bioavailability 90%
	Cladribine [16] (n = 1)	NR	No local toxicity
	Omacataxine [16] (n = 1)	NR	12 hr infusion studied
			**Endocrine**
**Grade A (strong evidence)**	Hydrocortisone [16, 24] (n = 5)	NR	Continuous less adverse effects than oral; higher QOL scores; no bone density
**Grade B (weak evidence)**	Calcitonin [16] (n = 1)	NR	Same bioavailability as IM
	Dexamethasone [16] (n = 1)	NR	Continuous infusion tolerated better than bolus
	Pamidronate [48] (n = 1)	A	Decreased reactions seen versus IV
	Parathormone [16] (n = 1)	NR	Showing clinical efficacy with low side effects but still in trials.
			**Gastrointestinal**
**Grade B (weak evidence)**	Cyclizine [16] (n = 2)	NR	
	Esomeprazole [16] (n = 1)	NR	
	Granisetron [16] (n = 1)	NR	
	Metoclopramide [16,62] (n = 2)	NR, A	Historical successful use in palliative settings but high adverse event profile with the medication regardless of route
	Omeprazole [16] (n = 1)	NR	Diluted in 100ml and administered over several hours
	Ondansetron [16,62] (n = 5)	NR, A	Low pH—slow infusions are better tolerated
	Palonestron [16] (n = 1)	NR	
			**Monoclonal Antibodies**
**Grade A (strong evidence)**	Trastuzumab [16] (n = 4)	NR	Local effects reported occasionally to frequent with some serious but preferred over IV due to IV safety concerns
**Grade B (weak evidence)**	Alemtuzumab [16] (n = 2)	NR	Rare local reactions
			**Neurologic**
**Grade B (weak evidence)**	Flunitrazepam [16] (n = 1)	NR	Effective for agitation, insomnia and dystonia
	Levomepromazine [16] (n = 3)	NR	Studied as 24hr continuous infusion
	Levetiracetam [16, 61] (n = 3)	NR	80% seizure controlled but no kinetics studies
**Inconclusive**	Clonazepam* [16] (n = 1)	NR	
	Midazolam* [16] (n = 1)	NR	Same bioavailability as IV, quickly reversible with flumazenil
			**Pain Management**
**Grade A (strong evidence)**	Hydromorphone [48] (n = 3)	A	Comparable to IV as a PCA
	Ketamine [16, 30] (n = 7)	NR, A/P	
	Morphine [48] (n = 25)	A	Comparable efficacy to IV and less side effects
		
**Grade B (weak evidence)**	Baclofen [16] (n = 1)	NR	Prevents withdrawal during tapering
	Buprenorphine [33] (n = 1)	A/P	14% local adverse events but often described as “best tolerated NSAID”
	Ketorolac [16] (n = 2)	NR	
	Methadone [16] (n = 4)	NR	High dose led to frequent local effects and half-life unpredictable
	Tramadol [16] (n = 1)	NR	Local effects and nausea but less than oral
**Inconclusive**	Diclofenac* [16] (n = 2)	NR	
			**Other Medications**
**Grade A (strong evidence)**	Immunoglobulin G [16, 29, 31] (n = 33)	NR, A/P	Well tolerated and good efficacy if dose adjusted for bioavailability; increased QOL scores reported
	Treprostinil [26, 37] (n = 2)	A, A/P	For treatment of patients with moderate symptoms; moderate evidence of safety and efficacy; however short trial follow-up
	Desferrioxamine [15, 25] (n = 15)	A/P	Remains the established treatment to reverse cardiac dysfunction due to iron overload, Weak evidence to determine if oral is as effective and has higher adherence.
**Grade B (weak evidence)**	Furosemide [16, 48] (n = 7)	NR, A	30 min delay to effect versus IV onset of 3–5 minutes
	Potassium [16] (n = 1)	A	Small sample size and slow absorption but good local tolerance for infusion but contraindicated as a bolus
**NOT Recommended**	Terbutaline [63] (n = 4)	A	Low evidence of efficacy but FDA states poor maternal outcomes
**Inconclusive**	Acetylcysteine [36] (n = 1)	A	No benefit seen but low power and poor design; studied as antioxidant in ALS
	Magnesium [16] (n = 1)	NR	10% solution continuous infusion

+ Age of participants, A -Adults, P -Pediatrics, A/P -Adults, Pediatrics, NR -Not reported (Data assessed in aggregate; discretion needed and further studies recommended when applying to specific age groups).

*Only studied in healthy volunteers;

^1^ Cmax—maximum (or peak) serum concentration that a medication achieves in a specified area of the body after the dose has been administrated and before the administration of a second dose;

^2^ Tmax—time after administration of a drug when the maximum plasma concentration is reached; when the rate of absorption equals the rate of elimination. NOTE: Number studies cited are based on the content of the SRs reviewed and more data may be available; n–number studies. Grade (A): strong evidence of adequate quality describing desirable effects outweighing undesirable effects and values, preferences and patient experience supports its use; Grade (B): weak evidence, although not of high quality, support its use, with desirable effects appearing to outweigh undesirable effects, although this is not as clear; Grade (Inconclusive): insufficient evidence to make a recommendation in less than 50% of studies (adapted from Aromataris et al., [18]).

#### Effectiveness

In general, medications administered subcutaneously are absorbed slower than given IV; however, can reach similar plasma levels [16]. Medications in the reviews were generally found to be equivalent in efficacy as when given via other routes such as intravenous or orally. Pain medications have the most extensive reported SCI use. Extensive evidence was reported for the use of subcutaneous morphine compared to intravenous and multiple other routes of administration demonstrating good effectiveness and safety (Table 5). Subcutaneous infusions were reported compared to epidural and intravenous of various opioids finding them to be equivalent or equivalent in efficacy, safety and acceptability [48].

For pain management, SCI of morphine was compared with epidural infusions and found to be acceptable and effective with fewer hypotension concerns with subcutaneous than epidural [59, 60]. Favorable subcutaneous use has been reported with baclofen, buprenorphine, hydromorphone, ketorolac, ketamine, methadone, morphine, tramadol [16, 48]. Both intravenous and epidural morphine patient-controlled analgesia (PCA) were evaluated in 5 studies and reported to be comparable [48].

Two antibiotics (ceftriaxone and ertapenem) were found to have similar antimicrobial effect, although variances did occur with Tmax and Cmax values and need to be taken into consideration if time to maximal plasma concentration and serum concentration are important clinically [16, 44]. For diuretics, overall clinical results were found to be adequate when compared to intravenous administration of the same medication, although onset of action may be delayed which can limit use in emergency situations [16, 48]. For endocrine medications, efficacy evaluated by return to expected circadian rhythms for cortisol was acceptable with less adverse events reported than other routes of administration [16, 24, 48]. Anti-epileptics were evaluated based on blood levels and seizure control and found to be adequate [16, 61]. Anti-nausea medications and neuroleptics were evaluated based on symptom improvement. Anti-nausea medications were found to show decreases in nausea and vomiting ranging from 62–93% depending on medication and patient population [16, 62]. One matched cohort trial found metoclopramide to be significantly less tolerated than ondansetron when administered by continuous subcutaneous infusion (4.4% versus 31.8%, p<0.001) [62].

Midazolam is considered the safest choice for SCI as it is the only water-soluble benzodiazepine and it has a fast onset, good tolerance and short half-life while still being reversible by antidote flumazenil even after administration [16]. Chemotherapy agents and biologics, such as Trastuzumab, administered by SCI are limited but those reviewed were found to have good bioavailability, similar terminal half-lives and found to be equivalent or non-inferior in response rates to intravenous routes [16, 38]. SCI of desferrioxamine has been shown to be effective with high levels of adherence and still considered a well-established method of iron chelation [15,25].

SC immunoglobulin (SCIG) infusion was found to achieve acceptable trough levels and reported effectiveness similar to IV infusion [29]. SCIG was also associated with higher health related quality of life and reduced infection rates which both are used to evaluate effectiveness of immunoglobulin therapy.

SCI of treprostinil for pulmonary hypertension was compared to placebo and found to be significantly more effective with minimal and tolerable side effects but no reviews included a comparison to intravenous [37]. The reviews showed statistically significant improvement in mean pulmonary artery pressure (mPAP), pulmonary vascular resistance (PVR), right arterial pressure (RAP) and cardiac index (CI). Changes in exercise capacity and dyspnoea were statistically significant but uncertain clinical significance. Quality of life scores reported higher in study group than placebo. While intravenous studies against placebo showed greater magnitude of clinical benefit versus the magnitude seen between subcutaneous and placebo, the intravenous studies had a 12–25% reporting of serious adverse events (e.g. sepsis, pulmonary embolism, hemorrhage) with only minor events reported in the subcutaneous group. While reported in both the placebo group and the treprostinil group, infusion site pain and redness was reported as statistically significant for treprostinil but bleeding and bruising was not reported to be statistically significant [37]. Adverse events related to the medication such as diarrhea, jaw pain, vasodilation, and edema were statistically significant compared to placebo but very similar among routes of administration. While mortality was not a reported outcome in the subcutaneous trials included in the reviews, the improvement in RAP and CI reported are considered reliable indicators of survival for pulmonary hypertension per the European Society of Cardiology [26].

#### Safety

Most commonly reported adverse events were local tissue reactions including redness, pruritus, and itching at the administration site (Table 5). Overall, the reviews reported adequate to good tolerance for the administration of the medications reviewed, once side effects attributed to the medication via any administration route were eliminated. However, caution must be taken, and patients advised to report, any adverse events, as studies have been very limited for most medications. Specific infusion rates and diluents vary based on medication and patient characteristics and could potentially impact tolerance. For example, in the treprostinil study, a placebo of diluent only was used and some site reactions still reported by placebo group but analysis not provided as to whether this occurred at lower doses or only with titration [26, 37].

Medications contraindicated for subcutaneous infusion administration include severe irritants such as higher supplements of potassium chloride (contraindicated as bolus but slow rates may be tolerated), lipo-soluble drugs such as diazepam which can precipitate in the skin, and medications known to cause fat necrosis such as chlorpromazine and prochlorperazine [16].

In cases of high osmolarity medications, such as subcutaneous immunoglobulins (SCIG), the subcutaneous route was considered safer as the infusion had less cardiovascular risks associated with the infusion. Reactions were more limited with the SCIG infusion versus the intravenous route with local skin whelps and irritation being the most frequent complaint. Specifically, in one review, 15 studies (376 patients) reported comparing adverse events during treatment for patients with Primary Antibody Deficiencies (PAD) with SCIG and IVIG, respectively [29]. The calculated odds ratio of 0.09 (0.07–0.11; p<0.001) indicates a significant preference of SCIG over IVIG because of a decrease in systemic adverse events. The data analysis clearly demonstrates that SCIG therapy, independent of the rate of infusion (standard, “rapid” or “express” administration), is associated with a high incidence of local reactions (44.7% for SCIG 10%, 92% for SCIG 16%, and 100% for SCIG 20% vs. 32% of IVIG 10%).

#### Acceptability

In one large systemic review, 5 of 94 studies reported preference for subcutaneous over IV with reasons varying from less side effects, to less time involved, less pain with access/administration or overall comfort [16]. SCIG patients also reported acceptability and a preference for subcutaneous. In one review specifically designed to evaluate patient acceptance of SCI versus intravenous medication administration, acceptability or preference for subcutaneous was found in 4 of 6 studies, ranging from 44–91% preferring SCI over intravenous with no significant difference found in one study and one showing a small preference for intravenous [38]. Reported reasons for the SCI preference included home/independent administration, convenience, less administration discomfort, and no end-of-dose weakening. Reasons of the group that preferred IV was not reported.

#### Efficiency

Few studies specifically reported on resource savings. In the review of clinical efficacy for diuretics, hospitalization of those patients with poor venous access was avoided in 83–93% cases [16]. One report on SCI of anti-emetics found it to be cost prohibitive versus other therapies (not versus other routes of administration) but since the time the article was published, the medication cost has drastically changed [62].

Shifting from hospital based IVIG treatment to home based SCIG is reported as “25–33% of the annual costs (US$11,000 per patient per year) in Sweden, 50% of the annual costs (€17–77 million) in Germany, CA$2,000 per patient per year in Canada (total cost savings CA$ 9,000,000/y), 25–50% of the annual costs in France, and $2,000–5,000 per patient per year in the US” ([29], p.188).

### Summary of findings

This synthesis of systematic review data supports the overall strong recommendation for the use of subcutaneous hydration infusions in the older adult population, due to its strong safety, efficacy, and acceptability profiles as summarized in Table 5. Data synthesis has led to an overall weak recommendation for subcutaneous hydration for pediatric patients and inconclusive for the palliative care/terminally ill population. Our analysis concurs with the findings of Bruera and colleagues, [40] who suggest that the use of SC hydration should be based more on the patient’s comfort than on providing optimal hydration. No recommendation can be made for the young and middle age adults, as no studies in the reviews addressed this population.

Overall ratings for medications were based on the success and strength of studies evaluating subcutaneous administration (Table 5). Ten medications have a Grade A recommendation, but 28 medications were assessed at B grade, indicating that more high quality studies are needed. Inconclusive was assigned to eight studies in which only healthy volunteers were used, as effectiveness cannot be evaluated, and safety could be biased. A designation of “not recommended” was assigned (amikacin, gentamicin, netilmicin, and terbutaline) when risk was found to outweigh the benefit or patient harm (e.g., skin necrosis) occurred in studies. Due to data aggregation and/or lack of reported data in the original reviews, we were unable to separate recommendations by population (e.g., children and adults).

## Discussion

Subcutaneous therapy has been shown to be safe, effective, acceptable and efficient as a treatment modality for mild to moderate dehydration in the older and pediatric population when oral therapy is not feasible or fails. This is in accordance with the Emergency Nurses Association recommendation of the use of SC hydration as an alternative to peripheral IV insertion for the mildly to moderately dehydrated pediatric and elderly patients (moderate level of recommendation with moderate quality of evidence) [64]. Slesak and colleagues [53] concluded from their RCT of 96 elderly adults that SC hydration is “far superior” in confused patients and in those with difficult IV access, also supported by other RCTs [43, 50]. It is also particularly useful in older patients with cognitive impairment, with rehydration shortening the duration of delirium [43]. Intrascapular placement also reduces the risk of intentional dislodgement of the device if the is patient prone to pulling intravenous catheters out.

Data is inconclusive yet to the use of SC hydration in the palliative care population. A small prospective study further explored SC hydration in palliative care, demonstrating that family caregivers and hospice nurses can administer SC hydration at end of life at home with minimal burden, equipment or technical support [65]. A qualitative review found that symptomatology was the most mentioned determinant of whether to hydrate or not, while the main criticisms were the potential side effects and difficulty in withdrawing hydration [66]. Findings from an RCT of significant efficacy, as well as efficiency, has led two authors to recommend its use in pediatric emergency departments [16, 50]. A review of SC hydration in neonatal palliative population found no articles studying this population, although a survey of French nurses working in these two settings found 86% were interested in establishing protocols for the SC route for analgesia, anxiolysis or terminal sedation [67]. Although not actually studied in these settings, use of SC hydration in resource-limited settings may be recommended, particularly in the pediatric setting if intraosseous or nasogastric access is unavailable due to the ease of use and training requirements, compared to IV access [28, 50].

Rochon and colleagues [23] determined that the use of hyaluronidase to promote subcutaneous fluid is unresolved. The authors recommended using slower infusion rates to permit gradual fluid transfer into the intravascular space, rather than using hyaluronidase. Hypersensitivity reactions are reported to be rare [16]. A recent meta-analysis of hyaluronidase-facilitated hydration in children and adults found that infusion rates between SC and IV was not statistically significant, showing a difference of not more than 25 mL/hr faster with IVs, with the average being 7.3 mL/hour [34].

The study data reveal the following advantages of SC hydration over intravenous therapy: reduced catheter dislodgements, infection, and phlebitis (although more local reactions), clinical and biochemical improvements, less agitation related to the access device/infusion, increased patient/caregiver satisfaction, time and success in device placement, ease of use and number and cost of access devices and less staff time to start and maintain the infusion. A decision analytic model studying Spandorfer’s pediatric study [45] supports these findings, finding the mean cost of hyaluronidase-facilitated subcutaneous hydration $167 USD, with cost savings attributed to ease of access where IV access is difficult and with shorter emergency department stay [68]. A pharmacoeconomic analysis also found SC therapy to be less costly than IV, for both hydration as well as administration of drugs (including immunoglobulin and opioid infusions) [69].

It has also been noted (although not quantified) that avoidance of IV venipunctures may help preserve peripheral veins and thus indirectly facilitate IV therapy [53]. We therefore, encourage clinicians and organizations to add this route of hydration as consideration to the gold standard of IV therapy. While some posit that SC therapy is indicated for patients if they have difficult venous access [50], we suggest that this be considered as a front-line treatment, as a means to preserve patient’s vessel health.

Indications of the most appropriate route may include the patient’s severity of dehydration, mentation, venous integrity, and preference; the volume and rate of the required therapy; clinician competency and training, resource availability, care setting, and healthcare costs. Hyaluronidase may help facilitate the absorption of fluids; however further study is required to determine its safety, efficacy and efficiency. We were unable to retrieve any studies comparing outcomes related to site selection.

Limitations to the use of SC hydration include those who require high volumes or rapid rehydration or medications not appropriate for SC use. The maximum volume reported was two litres in a 24-hour period [43]. While most studies reported local site reactions were the most frequently cited complication, these tended to resolve without intervention. We were unable to provide recommendations for the young to middle-age adult due to the absence of systematic review data. An additional search of recently published studies revealed no data, thus identifying a significant area for future research.

Given the evidence supporting the safety and efficacy of subcutaneous therapy, we concur with researchers who call for standardized policies and procedures to help optimize both study results and uptake of this practice [46]. The Infusion Nursing Society Infusion Therapy Standards of Practice provide practice guidance at a high level [11]. Evidence shows that there is a need for clinicians to better understand how to safely and effectively implement this intervention [50]. Publication of in-depth clinical practical guidelines may also support further adoption of this alternative, albeit indirect, vascular access route to help preserve vessel health of patient who are living longer and requiring complex intravenous therapy.

Lack of training and organizational protocols and unawareness of the subcutaneous route for the administration of fluids is a barrier to use [50, 66]. In the pediatric study of SC hydration, the lack of organizational protocols and staff awareness limited its usage and hence available outcome data. Uptake of this practice would also be supported by further evidence comparing the types and dwell times of SC devices, sites, and mode of delivery (e.g., pump vs. gravity), which our study reveals variability. Although many of the included studies used a metal needle, studies comparing plastic cannula to butterfly needles showed a strong preference for the plastic cannula, finding a mean difference of 6.7 days in site duration (p = .0009) and patient and nursing preference (p = .00002 and .0002 respectively) in one study and 93.5 hours dwell versus 42.8 hours IV (p = .0002) [70, 71]. This evidence is further supported by the Infusion Standards of Practice recommending against the use of metal needles [11].

The investigation of the use of potassium chloride in hydration solutions was limited to three studies [16, 23, 43]. Given the irritant properties of this medication, the authors anticipated a higher rate of local site reactions than reported. These studies suggest potassium chloride may be used as a SC hydration additive; however, we concur with Rochon and colleagues [23] that it be used cautiously until further evidence is available.

The main strength of this study is that it has synthesized the evidence from a large number of systematic reviews that have investigated SC infusion for hydration and drug therapy to provide a comprehensive understanding of this intervention important to a wide range of healthcare professionals (pharmacists, nurses, medical officers). A limitation of this study includes the potential for missing some systematic reviews. As our intervention was SC infusion there may have been drugs that would not have been captured as studies reported on the effectiveness of a specific medication, not how it was administered and therefore the title and abstract did not indicate SC as an administering route and thus excluded from full text review. It is also possible that the quality of evidence may be higher; however, the lack of reported methodological details led to downgrading of the overall confidence in results. Investigation of alternate routes was beyond the scope of this study; however, nasogastric and intraosseous rehydration has been shown to be effective in the pediatric population [28].

The authors found it surprising that there were so few reported studies looking at subcutaneous administration of medication as an alternate route. A recently published narrative review of subcutaneously administered antibiotics, however, confirmed the acceptable pharmacokinetic and pharmacodynamics data supporting the administration of ceftriaxone, ertapenem, and teicoplanin [72]. Of note, many medications are not officially approved by regulatory/licensing authorities via this route as large controlled studies are lacking currently and the expense of these studies outweighs the value in many cases. Therefore, individual case reports and small studies must be evaluated to decide if this route in an appropriate option.

Unfortunately, limited studies have been reported and included in systematic reviews evaluating the use of medications as subcutaneous infusions. While some have been studies as injections, the data on infusions is very limited in scope with the reports often not providing detail on the mechanisms of administration such as duration, rates, dilutions, diluents, access devices, etc. Several medications were only studies in healthy volunteers, where disease specific concerns don’t exist or in the palliative care setting where advance illness may skew the assessment. Many of the medications reported in this review need further evaluation with more controlled studies, broader populations and stronger evaluations. Additionally, the strength of the evidence is lacking in most reviews. Many reports are limited to case studies or retrospective reviews and not well controlled.

Limited studies included in systematic reviews looked at common therapies administered intravenous and subcutaneous including anti-emetics and treatments for pulmonary hypertension. While some studies did compare SC to oral or other therapies, the head to head comparison of the subcutaneous alternate route was lacking. The value of these studies shows safety and efficacy against placebo or oral but greater value would come from direct comparisons with intravenous so it would be easier to assess if intravenous and the associated risks could be avoided if oral is not an option.

Subcutaneous administration of opioids can be advantageous. Subcutaneous, like intravenous, avoids the first-pass effect of hepatic metabolism experienced with oral administration, which often limits the effectiveness and increasing adverse events from metabolites.

A trend was seen toward patient preference for subcutaneous infusions of chronic medications, such as SCIG. This appears to be influenced by ease of administration and low adverse events leading toward more patient independence and less interruption to schedule to receive the medication. Even when considering the additional doses required for SCIG, this preference was still found among many patients [38].

## Conclusions

The greatest strengths of SC therapy appear to be related to the ease of use and cost-effectiveness of this mode. Given the favourable outcomes in relation to effectiveness, safety, acceptability, and efficiency in the older and pediatric population, we suggest that subcutaneous access be considered alongside intravenous therapy as an alternative to oral hydration and some medications. Vascular access device selection algorithms do not currently include subcutaneous devices [10, 73], which we argue is an indirect access device. Inclusion of subcutaneous access devices in these algorithms may promote uptake of this access route and help preserve vessel health of our patients. Other opportunities for research which would help substantiate the practice of subcutaneous therapy include: the optimal subcutaneous access device properties (e.g., gauge, length, metal/cannula, and dwell time), type of hydration solutions/medications and additives and infusion delivery system.

## Supporting information

S1 Table PRISMA checklist. (DOC)

S2 Table Search strategy used for systematic review. (DOCX)

S3 Table List of excluded studies. (DOCX)

S4 Table AMSTAR quality scores of included systematic reviews. (DOCX)

S5 Table Subcutaneous hydration: Indications and contraindications. (DOCX)

S6 Table Subcutaneous hydration outcome. (DOCX)
