# Supplementary material for: Subcutaneous hydration and medications infusions (effectiveness, safety, acceptability): A systematic review of systematic reviews
Source: PLoS One. 2020 Aug 24;15(8):e0237572. doi: 10.1371/journal.pone.0237572 (PMC7446806; doi:10.1371/journal.pone.0237572)
Supplement: S2 Table — (DOCX) [file pone.0237572.s002.docx]

## **S2 Table. Search strategy used for systematic review- Embase**

|  | |
| --- | --- |
| **Search** | **Result** |
| #1 'subcutaneous infusion'/exp OR 'subcutaneous infusion' OR 'subcutaneous therapy' | 98,955 |
| #2 'hypodermoclysis'/exp OR hypodermoclysis | 247 |
| #3 #2 OR #3 | 99,133 |
| #4 (#2 OR #3) AND [1990-2019]/py | 55,414 |
| #5 (#2 OR #3) AND [1990-2019]/py AND [humans]/lim AND [english]/lim | 23,995 |
| #6 (#2 OR #3) AND [1990-2019]/py AND [humans]/lim AND [english]/lim AND ([cochrane review]/lim OR [systematic review]/lim OR [meta analysis]/lim) | 390 |
