# Supplementary material for: Subcutaneous hydration and medications infusions (effectiveness, safety, acceptability): A systematic review of systematic reviews
Source: PLoS One. 2020 Aug 24;15(8):e0237572. doi: 10.1371/journal.pone.0237572 (PMC7446806; doi:10.1371/journal.pone.0237572)
Supplement: S3 Table — (DOCX) [file pone.0237572.s003.docx]

| **S3 Table. List of excluded studies** | |
| --- | --- |
| **Lead Author (date)** | **Reason for exclusion** |
| Anderson (2004), Barnes (2007), Benedict (2007), Colin (2020), Hackman (2006), Lopez (2010), Oliver (2018), Park (2011), Pershad (2010), Pinto (2013), Saganski (2019), Thomas (2015) | Not systematic review, as defined by study criteria |
| Anandhan (2018), Basurto Ona (2013), Bayram (2003), Constantino (2012), Das (2010), Deleu (2004), Durand (2020), Fernando (2016), Forbes (2018), Frank (1997), Helfand (2009), Hua (2018), Kirkpatrick (2007), Leathersich (2018), Lee (2019), Lu (2015), Luttropp (2019), Malling (2004), Mouterde (2011), Nachiyunde (2018), Ng (2020), Nicolatou (2013), Papatsonis (2013), Pawitan (2018), Rice (2018), Ridyard, (2016), Riera (2016), Robertson (2017), Sasaki (2013), Shabaninejad (2016), Sharma (2012), Smeets (2016), Song (2016), Stewart (2018), Tarabay (2016), Torre (2002), Vardi (2009), Venclauskas (2018), Viola (2009), Visvanathan (2015), Volkmann (2013), Walker (2005), Zhang (2017), | Not PICO of interest or subcutaneous data not separate from other interventions* |
| Akbari (2016), Andrade-Castellanos (2015), Anstey (2015), Baker (2018), Barnard (2007), Benkhadra (2016), Churchill (2009), Colquitt (2003, 2004), Cummins (2010), Farrar (2016), Farsani (2017), Fatourechi (2009), Furnary (2006), Gandhi (2008), Gane (2010), Garg (2010), Golden (2012), Harrison (2014), Jeitler (2008), Kaiserman (2012), Li (2018), Matsuda (2014), Mazer (2009), Meijering (2006), Misso (2010), Mukhopadhyay (2007), Ontario Medical Advisory Secretariat (2009), Pankowska (2009), Ranasinghe (2015), Roze (2015), Rubin (2010), Rys (2018), Wolff-McDonagh (2010), Yeh (2012) | Not PICO of interest- insulin |
| Danielsen (2017), Li (2018) | Full text not available |
| Pouvreau (2017), Cássia Quaglio (2018) | Not English language |
| * Includes studies in which duration of infusion or type of subcutaneous therapy (infusion/injection) was unclear | |
