# Supplementary material for: Subcutaneous hydration and medications infusions (effectiveness, safety, acceptability): A systematic review of systematic reviews
Source: PLoS One. 2020 Aug 24;15(8):e0237572. doi: 10.1371/journal.pone.0237572 (PMC7446806; doi:10.1371/journal.pone.0237572)
Supplement: S4 Table — (DOCX) [file pone.0237572.s004.docx]

## **S4 Table. AMSTAR quality scores of included systematic reviews**

| \| Lead author \| 1. 1. PICO \| 1. 2. A priori design \| 3. Study design selection \| 4. Lit Search+ \| 5. Duplicate study selection \| 6. Duplicate data extraction \| 7. Exclu-ded studies \| 8. Study charac--eristics \| 9. Risk of  bias  assessed + \| 10. Funding sources \| 11. Meta-analysis stat. analysis \| \| 12. Meta-analysis risk of bias \| 13. Results reflect risk of bias assessed + \| 14. Hetero-geneity discussed \| 15. Publi- cation bias discussed \| 16.  COI noted \| Overall Confi-dence^ \| \| --- \| --- \| --- \| --- \| --- \| --- \| --- \| --- \| --- \| --- \| --- \| --- \| --- \| --- \| --- \| --- \| --- \| --- \| --- \| \| Abolhassani \| Y \| N \| N \| Y \| Y \| Y \| N \| P \| Y \| N \| Y \| \| N \| N \| N \| N \| Y \| L \| \| Al Nofal \| Y \| P \| N \| P \| Y \| Y \| N \| P \| Y \| N \| \| Y \| N \| Y \| Y \| N \| Y \| M \| \| Barnes \| Y \| N \| Y \| Y \| Y \| Y \| Y \| Y \| Y \| Y \| \| Y \| Y \| Y \| Y \| Y \| Y \| H \| \| Bell \| Y \| Y \| N \| P \| Y \| Y \| Y \| Y \| Y \| Y \| \| N/A \| N/A \| Y \| Y \| N/A \| Y \| H \| \| Bredlau \| Y \| N \| Y \| P \| N \| N \| N \| P \| N \| N \| \| N/A \| N/A \| N \| N \| N/A \| Y \| CL \| \| Duems-Noriega \| Y \| N \| N \| Y \| N \| N \| Y \| P \| N \| N \| \| N/A \| N/A \| N \| N \| N \| Y \| CL \| \| Fisher \| Y \| Y \| Y \| Y \| Y \| Y \| Y \| Y \| Y \| Y \| \| Y \| Y \| Y \| Y \| N \| Y \| H \| \| Fonzo-Christe \| N \| N \| Y \| P \| N \| Y \| P \| P \| N \| N \| \| N/A \| N/A \| Y \| N \| N/A \| N \| L \| \| Forbat \| Y \| N \| N \| P \| Y \| Y \| N \| P \| Y \| N \| \| N/A \| N/A \| N \| N \| N/A \| Y \| L \| \| Fortin \| Y \| Y \| Y \| Y \| Y \| Y \| Y \| Y \| Y \| Y \| \| Y \| Y \| Y \| Y \| Y \| Y \| H \| \| Gomes \| Y \| N \| Y \| P \| Y \| Y \| N \| P \| N \| N \| \| N/A \| N/A \| Y \| Y \| N/A \| N \| M \| \| Gaudet \| Y \| Y \| Y \| Y \| Y \| Y \| Y \| P \| Y \| Y \| \| Y \| Y \| N \| Y \| Y \| Y \| L \| \| Good \| Y \| Y \| Y \| Y \| Y \| Y \| Y \| Y \| Y \| N \| \| N/A \| N/A \| Y \| Y \| N/A \| Y \| H \| \| Ker \| Y \| N \| N \| N \| Y \| Y \| Y \| Y \| Y \| Y \| \| Y \| Y \| Y \| Y \| Y \| Y \| H \| \| Lingman-Framme \| Y \| N \| Y \| Y \| Y \| Y \| N \| P \| Y \| Y \| \| N/A \| N/A \| Y \| Y \| N/A \| Y \| M \| \| Marikar \| Y \| N \| N \| P \| N \| N \| N \| P \| N \| Y \| \| N/A \| N/A \| N \| N \| N \| Y \| CL \| \| Orrel \| Y \| N \| N \| P \| Y \| Y \| Y \| Y \| Y \| N \| \| Y \| Y \| Y \| Y \| N \| N \| H \| \| Paramothayan \| Y \| Y \| Y \| Y \| Y \| Y \| Y \| Y \| Y \| N \| \| Y \| Y \| Y \| Y \| N \| Y \| H \| \| Reichmann \| Y \| N \| N \| N \| N \| N \| Y \| P \| N \| Y \| \| N/A \| N/A \| Y \| Y \| N/A \| Y \| CL \| \| Remington \| Y \| N \| N \| P \| Y \| Y \| N \| P \| P \| Y \| \| N/A \| N/A \| N \| N \| N \| Y \| CL \| \| Rochon \| Y \| N \| N \| P \| N \| N \| N \| Y \| N \| Y \| \| N/A \| N/A \| N \| N \| N \| Y \| CL \| \| Rouhani \| Y \| N \| N \| P \| N \| N \| N \| Y \| N \| Y \| \| N/A \| N/A \| N \| N \| N \| N \| CL \| \| Schmidt-Hansen \| Y \| N \| N \| P \| N \| N \| N \| Y \| Y \| Y \| \| n/a \| n/a \| Y \| N \| N \| Y \| H \| \| Stoner \| Y \| N \| N \| P \| N \| N \| N \| P \| N \| Y \| \| N/A \| N/A \| N \| N \| N \| Y \| L \| \| Turner \| Y \| N \| N \| P \| N \| N \| N \| Y \| Y \| Y \| \| N/A \| N/A \| Y \| N \| N \| Y \| H \| \| Wilhelm \| Y \| N \| N \| N \| N \| N \| N  *AMSTAR 2 items: Y= Yes; P= Partial; N= No; N/A= Not applicable; *Adapted from: Shea BJ, Reeves BC, Wells G, Thuku M, Hamel C, Moran J, Moher D, Tugwell P, Welch V, Kristjansson E, Henry DA. [21]. Critical quality flaws criteria: a) adequacy of literature search (excluding justification of language restrictions) ; b) risk of bias in individual studies being included; and c) consideration of risk of bias when interpreting result of the review. ^ Overall confidence in review results: *High* (H= no or one critical weakness; review provide accurate and comprehensive summary of results of studies); *Moderate* (M= More than one non-critical weakness but no critical flaws; may provide accurate summary of results of studies); *Low* (L= one critical flaw with or without non-critical weaknesses; may not provide an accurate and comprehensive summary of results); *Critically low* (CL= more than one critical flaw and should not be relied on to provide an accurate and comprehensive summary of available studies). \| Y \| N \| N \| \| N \| N \| N \| N \| N \| Y \| CL \| |  |  |  |  |  |  |  |
| --- | --- | --- | --- | --- | --- | --- | --- | --- | --- | --- | --- | --- | --- | --- | --- | --- | --- | --- | --- | --- | --- | --- | --- | --- | --- | --- | --- | --- | --- | --- | --- | --- | --- | --- | --- | --- | --- | --- | --- | --- | --- | --- | --- | --- | --- | --- | --- | --- | --- | --- | --- | --- | --- | --- | --- | --- | --- | --- | --- | --- | --- | --- | --- | --- | --- | --- | --- | --- | --- | --- | --- | --- | --- | --- | --- | --- | --- | --- | --- | --- | --- | --- | --- | --- | --- | --- | --- | --- | --- | --- | --- | --- | --- | --- | --- | --- | --- | --- | --- | --- | --- | --- | --- | --- | --- | --- | --- | --- | --- | --- | --- | --- | --- | --- | --- | --- | --- | --- | --- | --- | --- | --- | --- | --- | --- | --- | --- | --- | --- | --- | --- | --- | --- | --- | --- | --- | --- | --- | --- | --- | --- | --- | --- | --- | --- | --- | --- | --- | --- | --- | --- | --- | --- | --- | --- | --- | --- | --- | --- | --- | --- | --- | --- | --- | --- | --- | --- | --- | --- | --- | --- | --- | --- | --- | --- | --- | --- | --- | --- | --- | --- | --- | --- | --- | --- | --- | --- | --- | --- | --- | --- | --- | --- | --- | --- | --- | --- | --- | --- | --- | --- | --- | --- | --- | --- | --- | --- | --- | --- | --- | --- | --- | --- | --- | --- | --- | --- | --- | --- | --- | --- | --- | --- | --- | --- | --- | --- | --- | --- | --- | --- | --- | --- | --- | --- | --- | --- | --- | --- | --- | --- | --- | --- | --- | --- | --- | --- | --- | --- | --- | --- | --- | --- | --- | --- | --- | --- | --- | --- | --- | --- | --- | --- | --- | --- | --- | --- | --- | --- | --- | --- | --- | --- | --- | --- | --- | --- | --- | --- | --- | --- | --- | --- | --- | --- | --- | --- | --- | --- | --- | --- | --- | --- | --- | --- | --- | --- | --- | --- | --- | --- | --- | --- | --- | --- | --- | --- | --- | --- | --- | --- | --- | --- | --- | --- | --- | --- | --- | --- | --- | --- | --- | --- | --- | --- | --- | --- | --- | --- | --- | --- | --- | --- | --- | --- | --- | --- | --- | --- | --- | --- | --- | --- | --- | --- | --- | --- | --- | --- | --- | --- | --- | --- | --- | --- | --- | --- | --- | --- | --- | --- | --- | --- | --- | --- | --- | --- | --- | --- | --- | --- | --- | --- | --- | --- | --- | --- | --- | --- | --- | --- | --- | --- | --- | --- | --- | --- | --- | --- | --- | --- | --- | --- | --- | --- | --- | --- | --- | --- | --- | --- | --- | --- | --- | --- | --- | --- | --- | --- | --- | --- | --- | --- | --- | --- | --- | --- | --- | --- | --- | --- | --- | --- | --- | --- | --- | --- | --- | --- | --- | --- | --- | --- | --- | --- | --- | --- | --- | --- | --- | --- | --- | --- | --- | --- | --- | --- | --- | --- | --- | --- | --- | --- | --- | --- | --- | --- | --- | --- | --- | --- | --- | --- | --- | --- | --- | --- | --- | --- | --- | --- | --- | --- | --- | --- | --- | --- | --- | --- | --- | --- | --- | --- | --- | --- | --- | --- | --- | --- | --- | --- | --- | --- | --- | --- | --- | --- | --- | --- | --- | --- | --- | --- | --- | --- | --- | --- | --- | --- | --- | --- | --- | --- | --- | --- | --- | --- | --- | --- | --- |
