# Supplementary material for: Subcutaneous hydration and medications infusions (effectiveness, safety, acceptability): A systematic review of systematic reviews
Source: PLoS One. 2020 Aug 24;15(8):e0237572. doi: 10.1371/journal.pone.0237572 (PMC7446806; doi:10.1371/journal.pone.0237572)
Supplement: S5 Table — (DOCX) [file pone.0237572.s005.docx]

| **S5 Table. Subcutaneous hydration: Indication and contraindications*** | |
| --- | --- |
| **Indications** | **Contraindications** |
| - Mild to moderate dehydration [16, 23, 29, 35, 44] | - Precise fluid titration (e.g., heart failure and renal failure) [16, 43] |
| - Cognitive impairment states related to dehydration [23, 27, 43] | - > 3L fluid required per day [16, 26, 27, 35] |
| - Rescue rehydration after failed IV placement [28] | - Rapid fluid replacement [16, 26, 27] |
| - Repeated IV catheter dislodgements [23] | - Clinical evidence of poor tissue perfusion [27, 43] |
| - Dysphagia [23](Rochon et al., 1997) - Palliative support at end of life (opioid-induced delirium, hypercalcemia, thirst) [41] | - Medical conditions such as circulatory failure, congestive heart failure, pulmonary edema, gross edema, major electrolyte imbalances, such as hypernatremia (> 150 mmol/L), hyperosmolarity (> 300 mOsmol/kg), BUN/ creatinine ratio >25, hypoalbuminemia, severe thrombocytopenia and anasarca [16, 26, 35] |
|  | - Relative contraindications include coagulation disorders or hemorrhages (related to poor absorption due to peripheral circulation failure due to blood loss), edema, and altered skin integrity (e.g., near operative scars) [26] |
| *Cited as inclusion and exclusion criteria in methods sections of included studies | |
