# Supplementary material for: Subcutaneous hydration and medications infusions (effectiveness, safety, acceptability): A systematic review of systematic reviews
Source: PLoS One. 2020 Aug 24;15(8):e0237572. doi: 10.1371/journal.pone.0237572 (PMC7446806; doi:10.1371/journal.pone.0237572)
Supplement: S6 Table — (DOCX) [file pone.0237572.s006.docx]

## **S6 table. Subcutaneous hydration outcomes**

| **Study,** Citation, Design,  Comparator  (Quality) | **Efficacy** | **Safety** | **Acceptability** | **Efficiency** |
| --- | --- | --- | --- | --- |
| [45] RCT  SC vs IV  (NR) | Mean volume and duration of infusion: NS; 🡩 in emergency dept.: 28.7 vs 22 ml/kg (95% CI 0.7-11.8) and 🡫 in ED and inpatient: 31.2 vs 35.8 mL/kg (95% CI -16.2 to 10.7); weight changes: NS; insertion failures: 🡫 0/73 SC vs 2/77 IV, RR 14.79 (95% CI 2,87,76.08]; successful line placement: 🡩 (100% vs 78.7% IV, p<.0001); dehydration score: NS | At least 1 treatment-related event by 100% vs 90.7% IV (*p*=.01); infusion site reactions: 🡩 (74% vs 25.3 % IV); swelling: 🡩 (80.8% vs 21.3 % IV); edema 🡩 (6.8% vs 1.3%); infusion-site pain: NS (9) | Patient/ caregiver satisfaction: 🡩 (94.5% vs 73.3 % IV; p<.0001); difficulty inserting catheter 🡫 (RR 6.33, 95%CI 2.32 to 17.23; *p*= 0.0003); 🡩ease of administration (94.5% vs 65.3% IV; p<.0001); | Time from 1st catheter insertion attempt to start IV: 8.3 min. less; mean  total treatment time (1^st^ catheter attempt to end of infusion): 🡫 (1.3 vs 2.3 h IV; p<.001) |
| [53] RCT  SC vs IV  (High) | Clinical and laboratory changes: NS; SC and IV comparably effective; median volume: 750 mL/day, SC vs 1,000 mL/day (IV, p*=*.002); median duration of infusion: NS (6 days, range 1-36; vs IV 6 days, range 1-32; p=.33); insertion failures: median 0 (range 0-1) vs 0 (0-5) IV (p<.001) | Local side effects: NS; mild (29 SC vs 24 IV) and major (local edema, phlebitis , cellulitis, erythema and strong pain; 9 SC vs 8 IV); systemic side effects: NS (p=.68) for cardiac failure (2 SC vs 4 IV); hyponatremia (1 SC vs 2 IV; p*=*1.0) | Procedure discomfort, activity of daily living and nurses` feasibility scores: NS (MD 0.00, 95% CI -0.21 to 0.21; *p*= 1.0; p=.74; and *p=*.8 respectively); physicians’ score of feasibility of SC: significantly 🡩 (p<.01) | Time to insert IV catheter: 2 minutes longer (95%CI -4.80 to 244.80; p = 0.06); cannulae changed: MD 2 days (range .5-9) vs 2.8 (0.3-8.8) IV (p=.14) |
| [54] RCT  SC vs IV  (NR) | Biochemical restoration (serum urea, creatinine, osmolarity): NS; volume infused: NS (p=.92) | Local reaction (edema); catheter dislodgement higher in IV (RR 3.78, 95%CI 1.16 to 12.34; n = 67; p = 0.03) | NR | NR |
| [48] RCT  SC vs IV  (High) | Serum urea and creatinine changes: NS and (*p=.3 and p*=.5 respectively); efficacy and volume infused: NS (3.3L vs IV 3.6 L, p>.01) | Occasional local edema; local edema: SC, (2); agitation: ↑ in IV (37% vs IV 80%; p=.005); | Better tolerated by confused older adults | Number of cannulae: 34 vs IV 41 cannulae; average cost of cannulae: SC was 24% of IV; resiting of catheter: 13% vs 23% IV, p>.25) |
| [55] RCT  SC vs IV  (NR) | Clinical (improvement in general or mentation or oral intake) and biochemical restoration: NS (p=0.55)  Urea/creatinine ratio: significant reduction (0.14 +/- .013 to 0.119 +/- .013, p=.001); volume and duration of infusions: NS | Local reaction: low incidence (similar)  Catheter dislodgement: NS | NR | Number of catheter changes: NS |
| [52] RCT  SC vs IV  (NR) | Biochemical restoration and hydration: NS (osmolality p=.12); serum osmolality: NS | Minor local effects; erythema around needle: 2; hyponatremia: 2 vs 4 IV; bruising: 0 vs 1 IV | NR | Cost of cannulae: 🡫 (£4.93 SC vs £13.94 IV) |
| [56] RCT  (High) | Biochemical restoration: NS; smaller metabolic and hormonal changes (5)(5) | NR | NR | NR |
| [57] Pro- spective  (NR) | Clinical improvement: 77%; urea (*p* = 0.001), creatinine (p< 0.00 1) and sodium (p < 0.05): improvement | Local complications: 12%; fluid overload: 0 | NR | NR |
| [49] Pro-spective SC vs IV  ([Mod.-High) | SC and IV effective; general or clinical improvement: NS (57% vs 25% IV; p= 0.19); mentation: improved (12/23 patients with clinical improvement) | fluid-related complications and local reactions: 🡫 (p=0.04 and *p*=.19 respectively); 32 complications in 37% of patients vs 17 in 61% of IV patients; catheter dislodgment attempts: NS (p=.4) | NR | NR |
| **PALLIATIVE** | |  |  |  |
| [46] RCT  SC vs placebo  (Mod. High) | Symptoms of hydration: NS (p=.77); night-time delirium: worse in placebo at day four *(*p=.028), but not day seven; all other efficacy outcomes: NS | Survival: NS (p= .83) | Quality of life: NS | NR |
| [58] RCT  SC vs No Tx  (NR) | Chronic nausea post-24 hrs (p=.027) and relief of thirst: 🡫 significantly; delirium, mental status: NS | Erythema and local pain: NS (1, 36 hrs after treatment started); fluid retention symptoms | Anguish and mood: NS | NR |
| [47] Pro-  Spective  (High) | SC is useful and safe for terminally ill patients;  well maintained renal function; hyponatremia common | Edema: 3; local reaction to hyaluronidase: 2; site changes due to poor absorption (leaking and swelling): 47%; inflammation (37%), bleeding/bruising: 11% and unknown: 5%; SC is safe for hydrating terminally ill | Patient/family request to discontinue (reason not reported) | NR |

MD - median; Mod - moderate; NR - not reported; NS - not statistically significant; IV - intravenous; SC subcutaneous, 🡫 - decrease ; ↑ - increase
